# Metaheuristic Framework for Material Screening and Operating Optimization of Adsorption-Based Heat Pumps

**DOI:** 10.1021/acsomega.3c01797

**Published:** 2023-05-23

**Authors:** Beatriz C. Silva, Carine Menezes Rebello, Alírio E. Rodrigues, Ana M. Ribeiro, Alexandre F. P. Ferreira, Idelfonso B. R. Nogueira

**Affiliations:** † LSRE-LCM—Laboratory of Separation and Reaction Engineering—Laboratory of Catalysis and Materials, Faculty of Engineering, University of Porto, Rua Dr. Roberto Frias, Porto 4200-465, Portugal; ‡ ALiCE—Associate Laboratory in Chemical Engineering, Faculty of Engineering, University of Porto, Rua Dr. Roberto Frias, Porto 4200-465, Portugal; § Chemical Engineering Department, Polytechnic School Federal University of Bahia, Salvador 40210-630, Brazil; ∥ Chemical Engineering Department, Norwegian University of Science and Technology, Sem Sælandsvei 4, Kjemiblokk 5, Trondheim 7491, Norway

## Abstract

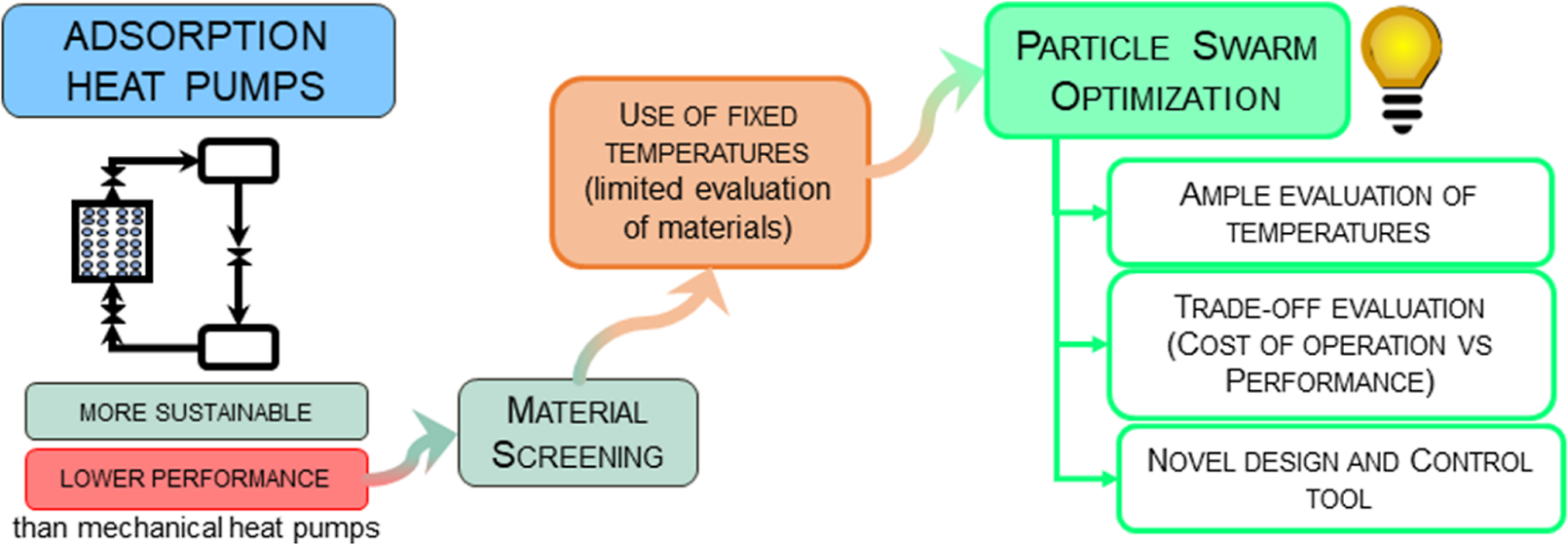

The current methods applied to material screening for adsorption-based heat pumps are based on a fixed set of temperatures or their independent variation, providing a limited, insufficient, and unpractical evaluation of different adsorbents. This work proposes a novel strategy for the simultaneous optimization and material screening in the design of adsorption heat pumps by implementing a meta-heuristic approach, particle swarm optimization (PSO). The proposed framework can effectively evaluate variable and broad operation temperature intervals to search for viable zones of operation for multiple adsorbents at once. The criteria for selecting the adequate material were the maximum performance and the minimum heat supply cost, which were considered the objective functions of the PSO algorithm. First, the performance was assessed individually, followed by a single-objective approximation of the multi-objective problem. Next, a multi-objective approach was also adopted. With the results generated during the optimization, it was possible to find which adsorbents and temperature sets were the most suitable according to the main objective of the operation. The Fisher–Snedecor test was applied to expand the results obtained during PSO application and a feasible operating region built around the optima, enabling the arrangement of close-to-optima data into practical design and control tools. This approach allowed for a fast and intuitive evaluation of multiple design and operation variables.

## 1 Introduction

Energy demand is continuously increasing, along with demographic growth. The energy consumption for domestic uses is about one-third of the global value.^1,2^ Most of that energy is spent on heating spaces or water for various ends.^2^

Usually, heating relies on electricity mainly generated by burning fossil fuels (ca. 40%).^3,4^ Due to environmental concerns, alternatives for the traditional heat production mechanism (such as vapor compression systems or conventional heat pumps) are being searched.

A possible alternative is the adsorption-based heat pumps. The operation of these heat pumps is based on thermal energy, reducing the electric power dependency. Furthermore, the heat can be produced based on renewable sources of heat (solar power), waste heat from industrial applications, or natural gas combustion as the least sustainable alternative.^5^ In addition, the adsorbent–adsorbate working pairs used in these pumps tend to be more sustainable than the working fluids employed in the vapor compression systems.^4,6^ Moreover, the lack of moving parts lowers the operation noise, which can be advantageous in domestic applications.^1,4,7^ At last, adsorption heat pumps have also been studied coupled with water harvesting since water is one of the most attractive adsorbates due to the lower environmental impact.^8^

However, they present some disadvantages, such as CO_2_ emissions, if the heat source relies on burning fossil fuels. Moreover, the heat production is not continuous when considering the most basic design. Furthermore, adsorption heat pumps have the propensity to present worse performance indicators than mechanical heat pumps since the operating temperatures are also lower.^1,4,7^

Some of the above-mentioned problems can be mitigated by employing a more advanced design or by searching the more adequate materials.^9−11^ Material screening is a technique that seeks to overcome the limitations of the adsorption heat pumps by comparing multiple adsorbent/adsorbate pairs to verify which one is the most promising in terms of performance. The work of Liu et al. (2022)^12^ is an example of using a pure thermodynamic model to select the top materials for cooling and heating applications, employing water as a working fluid. The adsorbent properties were also connected with their performance in both modes of heat pumping.

The usual approach used in the literature for material screening in heat pumps presents limitations. For starters, some approximations for the required parameters are used in the screening process, which may lead to a fallacious evaluation of materials. In addition, it is frequent to see fixed temperature limits for each step of the process or the variation of one of those temperatures, disregarding operating conditions where the performance might be similar. This has particular importance for industrial processes, where conditions might vary and affect the system’s performance.

A way to expand the range of temperatures in the study and avoid overlooking solutions that will lead to similar performances is to introduce material screening in the context of process optimization.

As reviewed by Venter,^13^ optimization procedures can be divided into two categories: local techniques and global ones.

Local techniques are typically based on gradients and are more efficient since fewer objective function (OF) evaluations are required to find the optimum. In addition to this fact, multiple design parameters can be used. However, these methods do not distinguish between global and local optima. Other disadvantages are the poor behavior when facing discrete variables and the complexity required for applying such methods.^13^

Global methodologies have the advantage of allowing local and global optima localization. One example of these methods is the evolutionary algorithms (EA).^13^

In EA, there is no need to calculate gradient information. The multiple points used allow for multiple starting conditions. These starting conditions can be random or predetermined using the Design of Experiments. The evolutionary methods are more consistent, providing a broader possibility of finding the global optimum and adaptability to discrete variables. On the downside, they demand more computational effort, limiting the size of the problems that can be solved through this type of methodology. Another disadvantage is the required tuning of the parameters (such as the size of the initial set of points) before the optimization procedure. The most typical models of this kind are the genetic algorithm, the particle swarm optimization (PSO), and ant colony.^13^ In more recent years, other methodologies have been unveiled among the EA, such as multi-verse optimizer.^14^

EA have given proof of their effectiveness in optimization, and improvement strategies for such algorithms are a recurrent theme in the literature. For example, surrogate-assisted EA arose as an alternative method for expensive multimodal optimization problems. The methodology proposed by Ji et al. (2021)^15^ consisted of a novel PSO strategy for solving the aforementioned problems (DSCPSO-EMM) using two populations of particles simultaneously to search the problem area for different modalities, creating a trade-off between the accuracy and the computational cost of the optimization. Multitasking or multifactorial algorithms are also presented as a solution by solving multiple optimization problems with the same population of particles. In the recent work of Ji et al. (2023),^16^ the aforementioned strategy was implemented within a multiple surrogate-assisted model PSO algorithm, and the results show an increased quality and quantity in the optimal solutions obtained when compared to other algorithms.

As reported by Su et al.,^17^ EA can interact with different particle issues such as data privacy, X-ray imaging segmentation,^18^ and medical-aided diagnosis,^19^ being more and more intertwined with other independent methodologies such as machine learning (ML). Feature selection is one of the examples of the synergy created between ML and EA. Hu et al.^20^ describes the optimization process to feature selection, considering fuzzy cost to ensure the balance between the feature cost and the system efficiency.

Another area where optimization is frequently used to solve concrete problems is chemical engineering,^21^ whether in food industries,^22^ energetic consumption optimization,^23^ or even cyclic process optimization.^24^

In fact, evolutionary methods of optimization have been used in heat pumping. Lee and Kung (2008)^25^ used a PSO algorithm to optimize the energy recovery in an indoor pool using discrete and continuous parameters. In the work of Rahman et al. (2013),^26^ the specific cooling power was also maximized based on the optimal cycle time using PSO. During the multi-objective optimization carried out by Li et al. (2019),^27^ the energetic, environmental, and economic impacts of a solar hybrid heat pump heating and cooling system were optimized by resorting to a genetic algorithm method.

However, no report was found of the employment of the adsorbent material as one of the variables in the optimization of adsorption heat pumps. Even when different materials are considered, the overall tendency in cyclic processes is to treat each material individually and then compare the optimal points for each adsorbent.^28^

Nevertheless, the use of the adsorbents as a discrete variable is starting to grow in this type of process.

In the recent work of Nogueira et al. (2022),^29^ the adsorbent material is treated as a discrete decision variable during a pressure swing adsorption process optimization using a modified version of the PSO algorithm. This modified version was named constrained sliding particle swarm optimization (CSPSO) and allowed for a compartmentalized search within the constraints and the defined variable limits.^30^ Furthermore, a feasible operating region (FOR) is built based on the Fisher–Snedecor test. This step allows the widening of the operating conditions to contemplate combinations of parameters that will result in a close-to-optimal performance. The authors of this work concluded that different materials could be employed in different conditions with similar performance despite their metric’s criteria.

Building an FOR is, thereby, a valuable tool in chemical engineering. The assurance that the productivity of a process will remain very close to the maximum for a certain range of values of the variables can help in the design of the process or even in process control operations.

This tool was introduced by Nogueira et al. (2019)^31^ in the sliding PSO method. It was later extended to constrained problems by Rebello et al. (2021a)^30^ in CSPSO for single-objective optimization and afterward expanded to a multi-objective optimization problem by Rebello et al. (2021b).^32^ In the last problem, the optimal points in the feasible region are grouped in clusters. Typically, the clusters divide the data into three regions: two where one of the objectives prevails and one region where there is a compromise between the two goals. The decision variables can also be represented in this cluster system, as demonstrated in the work of Rebello et al. (2022).^33^

All facts considered, there is room for improvement in the material screening in adsorption heat pumps. The present work aims to fill the gaps in this procedure by employing the material as a discrete variable within an optimization methodology and evaluating the different materials in a wider range of temperatures. For that, a PSO algorithm was implemented, considering that the best materials should have outstanding performance while keeping the energetic consumption to a minimum. Multiple optimization approaches were taken, namely, (a) the single-objective optimization of the performance, (b) a single-objective approach in which the OF is a weighted sum of the performance coefficient and the energetic cost of operation, and (c) a multi-objective optimization resulting in a Pareto Front juxtaposing both objectives.

Furthermore, feasible regions were built for assessing variables’ intervals that lead to similar performances to the most probable value (MPV) of the optima, within a given uncertainty. The said regions were outlined with a Fisher–Snedecor test to ensure the likelihood between the MPV of the optima and the points within the feasible regions.

Hence, a concise and comprehensive framework was developed for simultaneously optimizing and designing adsorption heat pumps, with the following contributions to the field.

a The application of PSO algorithms to the material screening procedure, presenting a tool for a systematic and extended evaluation and comparison of different materials, with a small computational effort.
b The assembly of innovative control and design tools based on the expansion of the optima points by the statistic test of Fisher–Snedecor.

## 2 Materials and Methods

### 2.1 Adsorption Heat Pump Model

Adsorption is the capture of an adsorbate, a given compound present in a stream, in an adsorbent, usually a solid material that interacts with and retains the adsorbate.^34^ In some applications, adsorption can be used as a heat source as it is an exothermal process. It is the operational basis for adsorption heat pumps, which produce heat based on the difference between the energy released during the adsorption phase and the energy consumed during the desorption phase.

**Figure 1:**
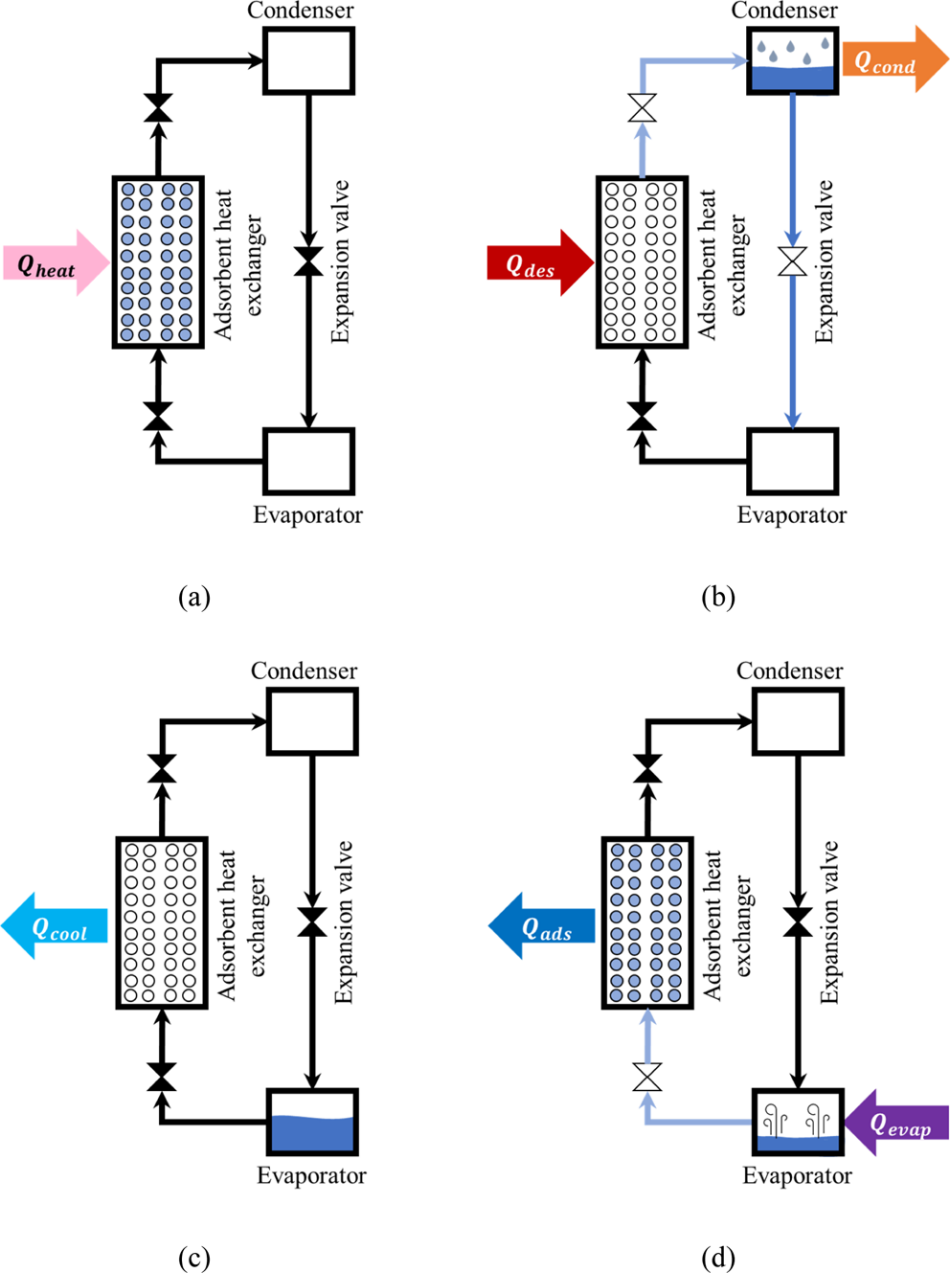
Working steps of adsorption heat pumps [isosteric heating (a), isobaric desorption (b), isosteric cooling (c), and isobaric adsorption (d)].

The most straightforward design for an adsorption heat pump consists of four main components: an adsorption heat exchanger, an evaporator, a condenser, and valves (including an expansion valve).^4^ The four working steps of adsorption heat pumps are well described in the literature as pictured in Figure 1.^1,4,7,35,36^

In isosteric heating, the adsorbent bed is isolated and heated to increase the pressure until the desired value is obtained. The valve that connects the adsorbent heat exchanger to the condenser is opened, leading to isobaric desorption.

During this second step, heat is provided to the system to promote the adsorbent regeneration by increasing the temperature and heating the adsorbent bed. The desorbed adsorbate flows to the condenser, releasing latent heat through the phase shifting at constant pressure.

The third step is isosteric cooling, where the adsorption heat exchanger is isolated and cooled to reduce the pressure back to the initial value, releasing sensible heat.

At last, the valve between the evaporator and the adsorption heat exchanger is opened, and isobaric adsorption begins. The working fluid in the evaporator receives heat from the environment, shifting into the vapor phase and later being adsorbed in the fixed bed. The heat produced during the adsorption process is then retrieved from the system.

The condenser and evaporator operating conditions are related to the liquid–vapor equilibrium and the temperature of the heating fluids used in the process.

**Figure 2:**
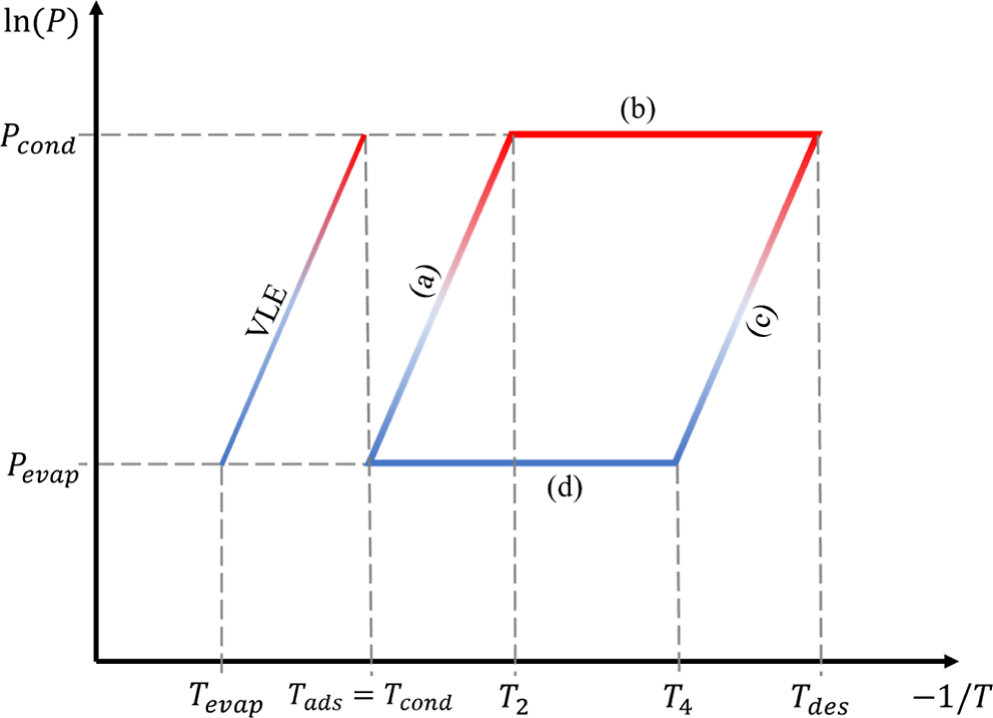
Diagram of ln(*P*) vs −1/*T* of an adsorption heat pump’s cycle operation [vapor liquid equilibrium, isosteric heating (a), isobaric desorption (b), isosteric cooling (c), and isobaric adsorption (d)].

Figure 2 is a schematic representation of the temperature and pressure limits as well as the heat exchanges between the system and the neighborhood.

For the simulation of the adsorption heat pumps behavior, a pure thermodynamic model was considered.^6,7,12,35−37^ The heat transferred between the heat pump and the neighborhood in each stage was calculated by the equations found in the Supporting Information.

### 2.2 Optimization Problem

#### 2.2.1 Optimization Goals

The coefficient of performance (COP) was used to evaluate the performance of the adsorbent-water working pair. The heating mode of operation was the focus of this work since it is the predominant cause of energy consumption.^2,3^ For the heating mode, this coefficient is defined as the amount of heat released from the system divided by the amount of heat provided to the system, as presented in eq 1.^4^
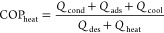
1

The higher the COP_heat_ value is, the more efficient the adsorption heat pump will be since more heat will be produced by unit of heat provided to the system. Therefore, the first objective of the optimization problem will be to maximize this function.

From the possible sources of heat, natural gas is the most common and available, with the downside of releasing carbon dioxide upon burning. Despite that disadvantage, it was considered that the heat source during the simulations represents a trade-off between energy consumption and productivity.

Therefore, a second OF was considered to minimize natural gas consumption. The said function relies on the cost of natural gas consumed during a cycle of operation. Because no dynamic interactions were considered in the model, the calculations were based on the heat required by the operation, as presented in eq 2.
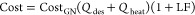
2

The cost of natural gas, Cost_GN_, was considered as 20.53 € GJ^–1^, the highest price paid during the second half of 2021 in Portugal (corresponding to the price of the lowest consumption band for domestic use).^38^ The loss factor (LF) corresponded to heat loss during the process and was considered to be 0.1.

The cost presented a reduced value when compared to the COP_heat_ value. To ensure that both OFs were treated with similar ponderation during the optimization problem, the cost function was multiplied by an impact factor, IF = 20. This factor guarantees that both OFs are in a similar order of magnitude. The adapted OF is presented in eq 3.
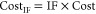
3

A single optimization of COP_heat_, as well as a single-objective approximation of the simultaneous optimization of COP_heat_ and Cost_IF_, followed by a multi-objective optimization of both functions, was performed. This last OF was not evaluated individually since it was predictable that the minimum value of the cost would result in the non-operability of the adsorption heat pump.

It is essential to mention that since no dynamic behavior was evaluated, both functions will be evaluated per cycle of operation, which may vary from material to material.

#### 2.2.2 Design of the Objective Function

The decision variables are essential in designing an OF. As the goal of this work is the simultaneous optimization of the process operating variables and material used, the decision variables chosen were *T*_evap_, *T*_cond_, *T*_des_, and the adsorbent material.

A second step in the OF design is the definition of the constraints. For the temperatures, the side constraints were based on a review article that summarized the most commonly used temperatures for each type of adsorbent–adsorbate working pair.^4^

The material is an integer variable introduced in the optimization problem. An approximation solved the continuous/discrete nature of this problem. A continuous variable was attributed to the material ranging from 0.51 to 10.49. Then, it was internally converted to an integer number by rounding it off, guaranteeing its discrete behavior. The materials considered during the optimization process and the corresponding attributed value are presented in Table 1. Water was considered as the adsorbate.

**Table 1:** Discrete Variable Values Attributed to Each Material

adsorbent	material value	adsorbent	material value
zeolite 5A	1	MIL-160 (Al)	6
zeolite 3A	2	zeolite 4A	7
MIL-100 (Fe)	3	MIL-125_NH_2_ (Ti)	8
Al-FUM	4	zeolite 13X	9
AQSOA FAM-Z02	5	CAU-10	10

The side constraints of each decision variable are presented in Table 2.

**Table 2:** Side Constraints of the Decision Variables

variable	lower bound	upper bound
*T*evap/K	280	303
*T*cond/K	309	365
*T*des/K	345	473
adsorbent material	0.51	10.49

However, not all the combinations contemplated by these ranges lead to a feasible solution. To avoid the consideration of such points, constraints were defined. In the optimization strategy, the constraints were assured by penalties. The penalization was marked by attributing the constant value of 100 to the OF(s) when the decision variables did not respect the constraints.

At last, the optimization problem can be summarized as presented in eqs 4–10:

Single optimization or
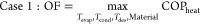
4
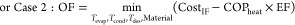
5with EF being the equalization factor for the OFs to present the same units. In this case, EF acquires the value of 1€.

Multi-optimization
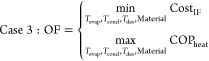
6

subject to
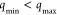
7
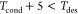
8
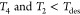
9
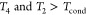
10

### 2.3 PSO Optimization

The previously presented optimization problem was solved using a PSO algorithm. The PSO is a meta-heuristic technique from the Artificial Intelligence field. It can be pictured as a group of individuals (particles) searching randomly in a certain space for the optimum solution to a proposed goal. Each particle evaluates its current position based on the value of the OF and determines its next step by analyzing its own history of those values and the history of the values for other particles. Iteration after iteration, the particles will get closer to the desired point of the OF. This process is accelerated through the combined information collected by different particles, allowing a faster exploration of the problem’s landscape.^39,40^

The algorithm used was based on the multi-objective feasibility enhanced PSO developed by Sinan Hasanoglu and Dolen (2018)^41^ and the CSPSO developed by Rebello et al. (2021a, 2021b).^30,32^ However, the restrictions were not applied as external conditions. Since comparison algorithms and the evaluation of benchmark functions had already been performed in the aforementioned papers, a similar analysis was not deemed necessary for the presented algorithm.

The parameter values have not suffered any particular optimization based on their influence in the process, being chosen from base literature. The alteration of the pack of adsorbents to be screened would also alter the optima for the parameters in PSO algorithm so that optimization would be a waste of computational effort. The only concern in the choice of parameter setting was that the values would make sense in the algorithm context.

Before the implementation of the PSO, the number of particles (), number of iterations (), number of OFs (*N*_OF_), number of decision variables (*N*_DV_), and respective decision variables’ side constraints (*x*_*n*,lower_ and *x*_*n*,upper_) should be defined in the system.

**Figure 3:**
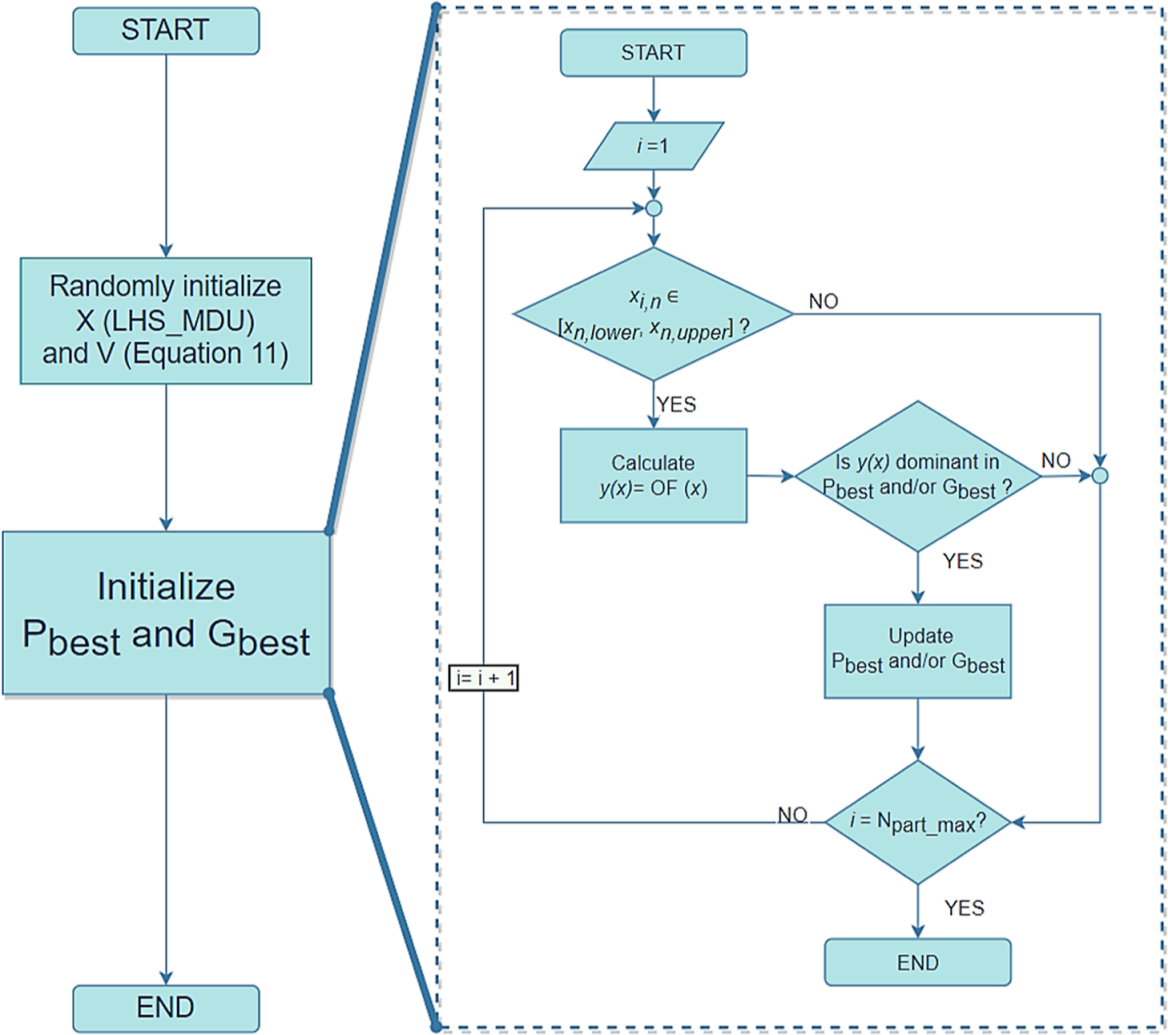
Initialization algorithm.

The initialization step is the first stage of this optimization strategy, where the positions (*x*) of the particles are randomly initialized through the latin hypercube sampling with multi-dimensional uniformity to ensure a good distribution of the initial particles in the problem field.^42^ The velocity (*v*) for each particle (*i*) for each decision variable (*n*) is initialized with eq 11. The initialization procedure is pictured in Figure 3.
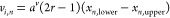
11

In this equation, *a*^*v*^ = 0.3 and corresponds to the initialization factor of velocity and *r* is a random number between 0 and 1.

The values of the position and velocity of the particles are stored on the matrices *X* and *V* of dimensions . For each particle, the corresponding values of the OF(s) are calculated [*y*(*x*) = OF (*x*)] and stored in a matrix *Y* with dimensions . The matrices *P*_best_ and *G*_best_ are also created to save the best positions and the corresponding values of the OF(s) of each particle and the global swarm, respectively. The actualization of these matrices is performed by the assessment of the dominance, where the values calculated in each step of the algorithm are compared to the points stored in *P*_best_ and *G*_best_^.^ The best values are saved in the matrices [both *x* and *y*(*x*)], and the values that are no longer “dominant” are eliminated from that data set.

The PSO procedure is presented to fit single-objective and multi-objective optimization since the main differences between both modes are the dimensions of the matrix *Y* and the number of objectives to be considered during the assessment of dominance.

After the initialization algorithm, the optimization step begins. A new velocity (*v*_*i*,*n*,*k*_) is calculated for each particle by eq 12 where a position from *G*_best_ and another from *P*_best_ are randomly chosen to be the guide positions ( and ), *r*_1_ and *r*_2_ are two random numbers between 0 and 1, *C*_o_, *C*_1_ = 2, and *C*_2_ = 2 are acceleration coefficients, and *k* is the current iteration.
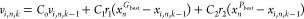
12

As the iteration number increases, the particles will get closer to the optimal point(s), so the velocities should decrease. To ensure that happens and to ease the system’s convergence, *C*_o_ is recalculated at each iteration with eq 13. The remaining coefficients are constant throughout the optimization process.
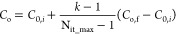
13with *C*_o,f_ = 0.4 and *C*_0,*i*_ = 0.9 being the final and initial values of *C*_o_ (*C*_o,f_ < *C*_0,*i*_).

The particle position is then updated, resorting to eq 14.
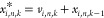
14

This new position should obey the upper and lower bounds defined for each variable. If this situation is verified, *x*_*i*,n_^*^ = *x*_*i*,*n*,*k*_. If not, the velocity of the particle is annulled (*v*_*i*,*n*,*k*_ = 0) and a new position is calculated. First, violation rates (*w*_L_ and *w*_U_) are calculated to evaluate the maximum limit trespassing between all the decision variables as shown in eqs 15 and 16. Then, the maximum violation rate [*w* = max(*w*_L_, *w*_U_)] is used to find the new particle position according to eq 17.
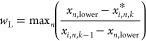
15
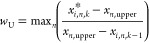
16
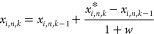
17

**Figure 4:**
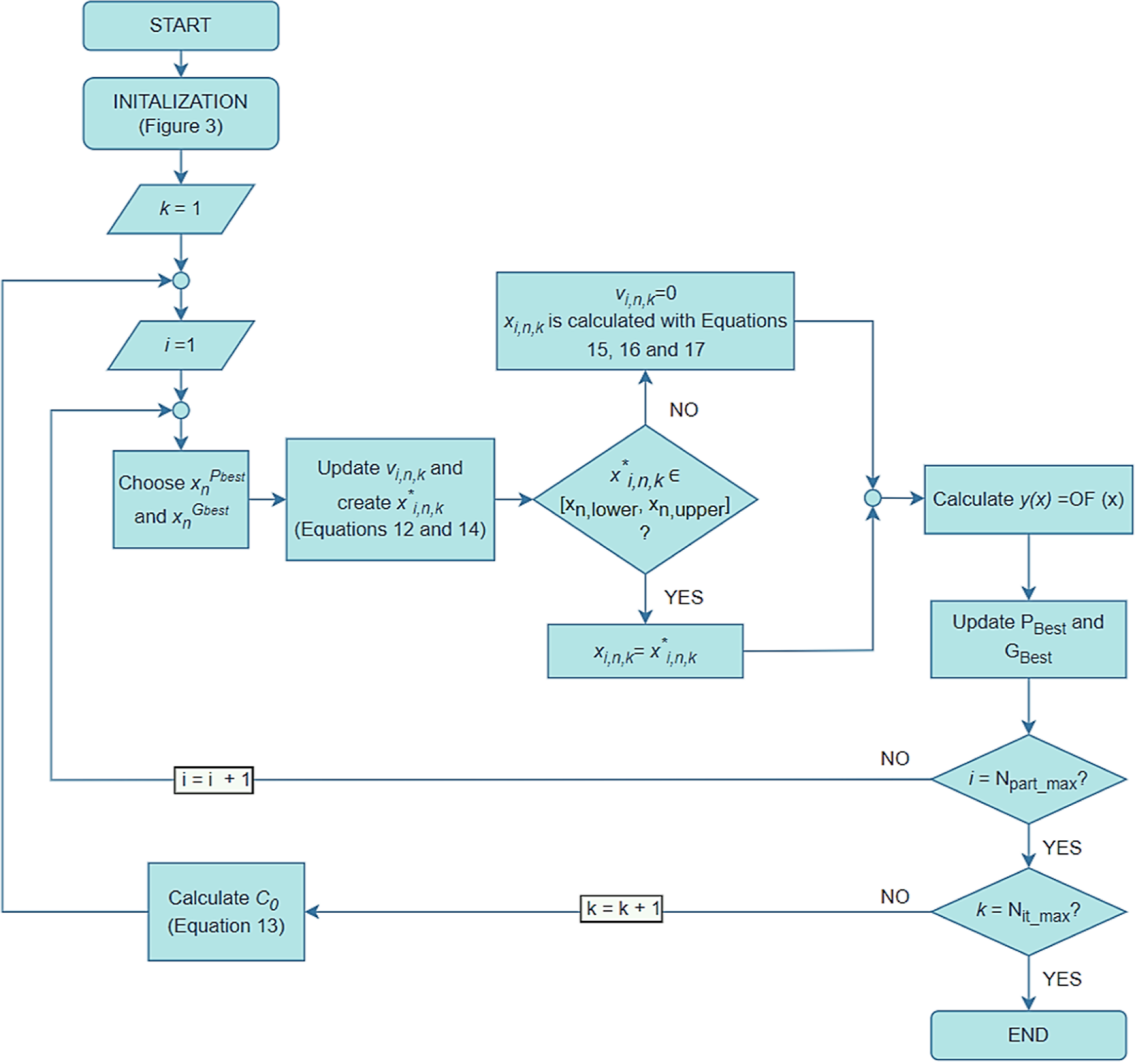
PSO algorithm.

The complete PSO algorithm is depicted in Figure 4.

At the end of the algorithm, it is possible to obtain a matrix *G*_best_ containing the optimal point (in single-objective problems) or the optimal Pareto Front (in multi-objective problems) and the decision variables that lead to those solutions. However, there might be close-to-optima solutions that are not contemplated in the optimal point/curve generated by the values in *G*_best_. Therefore, the values of the particles generated during the implementation of the algorithm were stored to determine the FORs.

### 2.4 Uncertainty Assessment of the Optimization Problem

With the particles generated during the PSO implementation, building an FOR containing close-to-optimal points was possible. The evaluation was based on a Fisher–Snedecor test, whose deduction was previously presented in the literature.^30,32,33^ Given a certain particle with the corresponding calculated value of the OF(s), the said particle will be part of the FOR if the condition in eq 18 is followed.
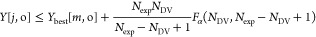
18with *N*_exp_ being the number of experiments considered, α the confidence interval, *Y* the matrix containing the global set of particles generated during the PSO algorithm with dimensions of , and *Y*_best_ the matrix containing the optimal values of the OFs with dimensions of [number of particles in *G*_best_, *N*_OF_].

**Figure 5:**
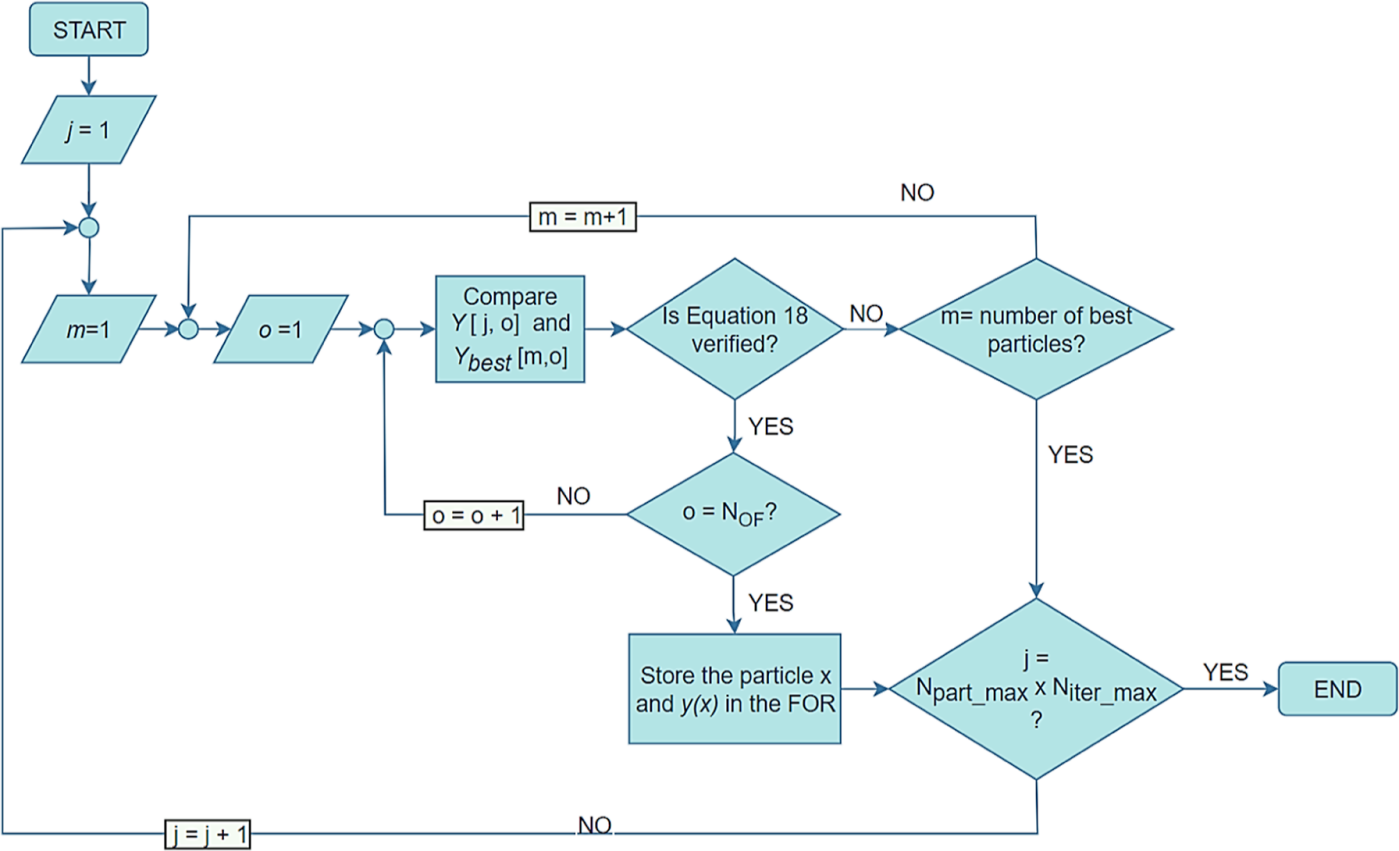
Construction of the FOR.

The methodology presented in Figure 5 was followed to examine the particles.

At the end of the evaluation, the stored points constitute the FOR. Since this test ensures that all the particles in the FOR are similar to the optima points obtained within a given significance level, no other statistical test was employed during the evaluation of results.

With the values in this region, it is possible to evaluate the uncertainty of the variable values that lead to the optimal solution. This assessment was made based on Type A Uncertainty, where the MPV for the decision variables (*x*_*i*_) and their confidence intervals (CI) were obtained based on eqs 19–21^43^
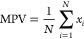
19
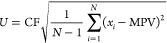
20
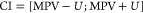
21where *N* is the number of points, U is the uncertainty, and CF is the coverage factor (considered equal to 1.96, associated with a level of confidence of 95%).

In multi-objective optimization, the FOR was divided into 3 clusters to evaluate which conditions lead to the prevalence of one of the objectives or the compromise between them. This clusterization action was performed using MATLAB’s cluster data function applying the Linkage based on the “weighted” criterion.^44^ This clusterization method is based on the mean distance between two clusters or a cluster and a point.^45^ At last, the uncertainty assessment was also performed in the cluster evaluation.

### 2.5 Simulation Scenarios and Computational Resources

The simulation and application of the PSO algorithm were implemented in a portable computer with 16 GB of RAM and a processor Intel(R) core (TM) i7-10750H CPU @ 2.60 GHz 2.59 GHz using the Spider 5.1.5. associated with Python 3.9.7 64 bit. The clusterization was performed with MATLAB version R2021a 64 bit in a server with 64 GB of RAM and two processors, Intel(R) Xeon(R) CPU E5-2650 v2 @ 2.60 GHz 2.60 GHz.

The parameters applied in each study case and the resulting run times are listed in Table 3.

**Table 3:** Parameters Used during PSO Optimization

parameter	case 1	case 2	case 3
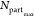	3000	4000	500
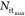	200	400	200
α	0.9995	0.9995	0.9999
*N*_exp_	3.000.000	3.000.000	1.000.000
run time/s	3480	12 858	368

The higher number of particles in single-objective optimization is justified by the need to have a larger number of particles in the convergence zone to ensure a good design of the confidence region. In the multi-objective problem, the number of particles had to be reduced; otherwise, the clusterization process would be compromised due to a lack of memory.

## 3 Results and Discussion

### 3.1 Particle Distribution Evaluation

Before analyzing each optimization result, a good practice is verifying the particles’ uniform distribution throughout the entire problem field. This is done to confirm the expected behavior of the algorithm, verifying if the particles traveled the whole landscape.

With that in mind, the OF values for all the particle position history during the optimization were plotted in function of the material and the three different temperatures. The resulting plots can be seen in Figure SI.22 in the Supporting Information for all the cases defined in the optimization problem.

The overall results reveal an excellent evaluation of the OF landscape.

For higher values of *T*_cond_, many particles were classified as unviable, which can be explained by the constraint that forces this variable to be 5 K lower than the desorption temperature and the fact that lower *T*_des_ was associated with better system performance, as will be discussed in the following sections.

Despite the strategy adopted for the penalization, it did not seem to be a trouble for the optimization procedure. The problem was therefore considered well-evaluated.

Furthermore, as Figure SI.23 in the Supporting Information demonstrated, the need to use a lower number of iterations did not prejudice the convergence of the particles toward the Pareto curve.

### 3.2 Single-Objective Optimization

#### 3.2.1 Maximization of COP_heat_

The optimization problem proposed in case 1 was solved following the previously described methods. An MPV of COP_heat_ equal to 1.86 was found. The best-suited material for maximum performance was material 3 [MIL-100 (Fe)].

With the application of the Fisher–Snedecor test, it was possible to build the FOR with a confidence level of 0.9995. The uncertainty was computed based on the MPV for each decision variable and the corresponding confidence interval. These results are displayed in Table 4.

**Table 4:** Most Probable Values for the Decision Variables and Respective Confidence Intervals for Maximization of COP_heat_

decision variable	MPV	CI
*T*evap/K	303	[301.4, 303]
*T*cond/K	309	[309, 309.7]
*T*des/K	345	[345, 354.7]
material	3	[3]; [8]

As it is possible to see, *T*_evap_ optimum was at the value of the upper bound, while *T*_cond_ and *T*_des_ converged to the lower bound. However, there is another suitable material that did not appear in the initial optimization result. The confidence interval is quite restricted for *T*_evap_ and *T*_cond_, but the values for *T*_des_ present a broader range. This is likely due to no consideration of the cost of the desorption process, allowing the use of a greater temperature despite the MPV being at the lower bound.

For reference, the Cost_IF_ for the optimal point obtained was 0.72 €.

**Figure 6:**
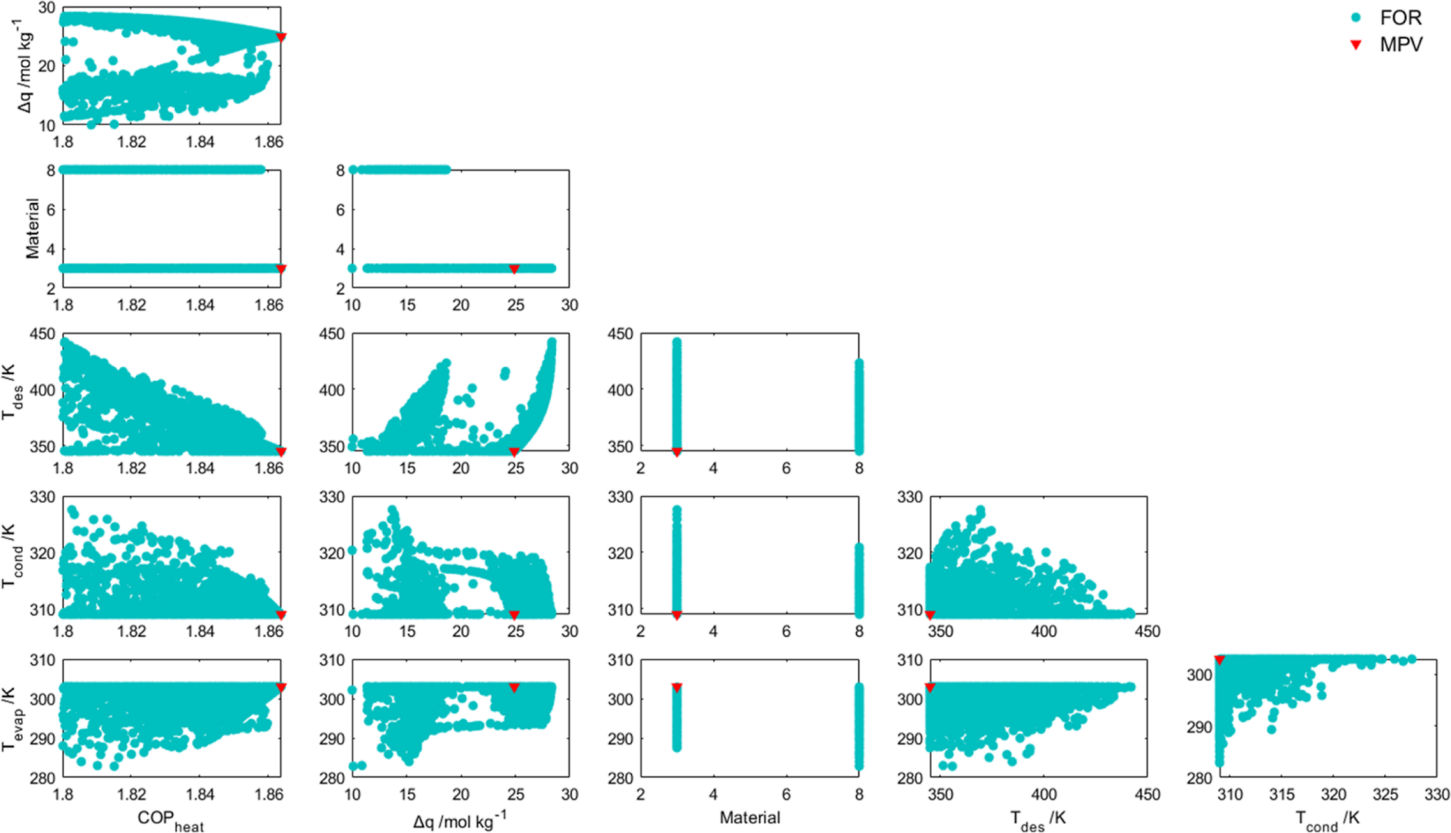
FORs obtained by the Fisher–Snedecor test for *T*_evap_, *T*_cond_, *T*_des_, Material, Δ*q*, and COP_heat_.

The FORs were plotted to better understand the different application zones for both materials, as shown in Figure 6. The value of Δ*q* was also added to the analysis because a higher Δ*q* leads to a higher amount of water condensed during the cycle, increasing the amount of heat released.

The plot of the material vs COP_heat_ shows that material 8 (MIL-125_NH_2_) presents a slightly lower performance than material 3 [MIL-100 (Fe)] even though both materials show the MPV of temperatures within their operation region defined by the optimal region of operation. This is due to the higher water adsorption capacity presented by MIL-100 (Fe).

An interesting fact about the Δ*q* is that it was not the highest value of this parameter to lead to the highest performance. This can be explained by the required temperature level to reach the maximum Δ*q* being relatively high (near 450 K), requiring more heat supplying and diminishing the COP_heat_. Furthermore, the maximum performance of MIL-125_NH_2_ is quite similar to that of MIL-100 (Fe) despite the lower Δ*q*. This is probably due to the lower isosteric adsorption heat of the MIL-125_NH_2_ leading to a lower need for heating during desorption phase.

The plots of temperature for this optimization basis do not show any correlation aside from the tendency for lower values of *T*_des_ and *T*_cond_ and higher values of *T*_evap_.

#### 3.2.2 Minimization of (Cost_IF_-COP_heat_ × EF)

The single-objective approach of the multi-objective optimization led to an optimal absolute value of 1.6 €. This corresponds to optimal values for Cost_IF_ and for COP_heat_ of 0.15 € and 1.75, respectively. Since a trade-off is considered, the cost of operation per cycle in the optimal point was reduced by 80%, making the heat production more affordable with the disadvantage of lowering the COP by 6%.

**Figure 7:**
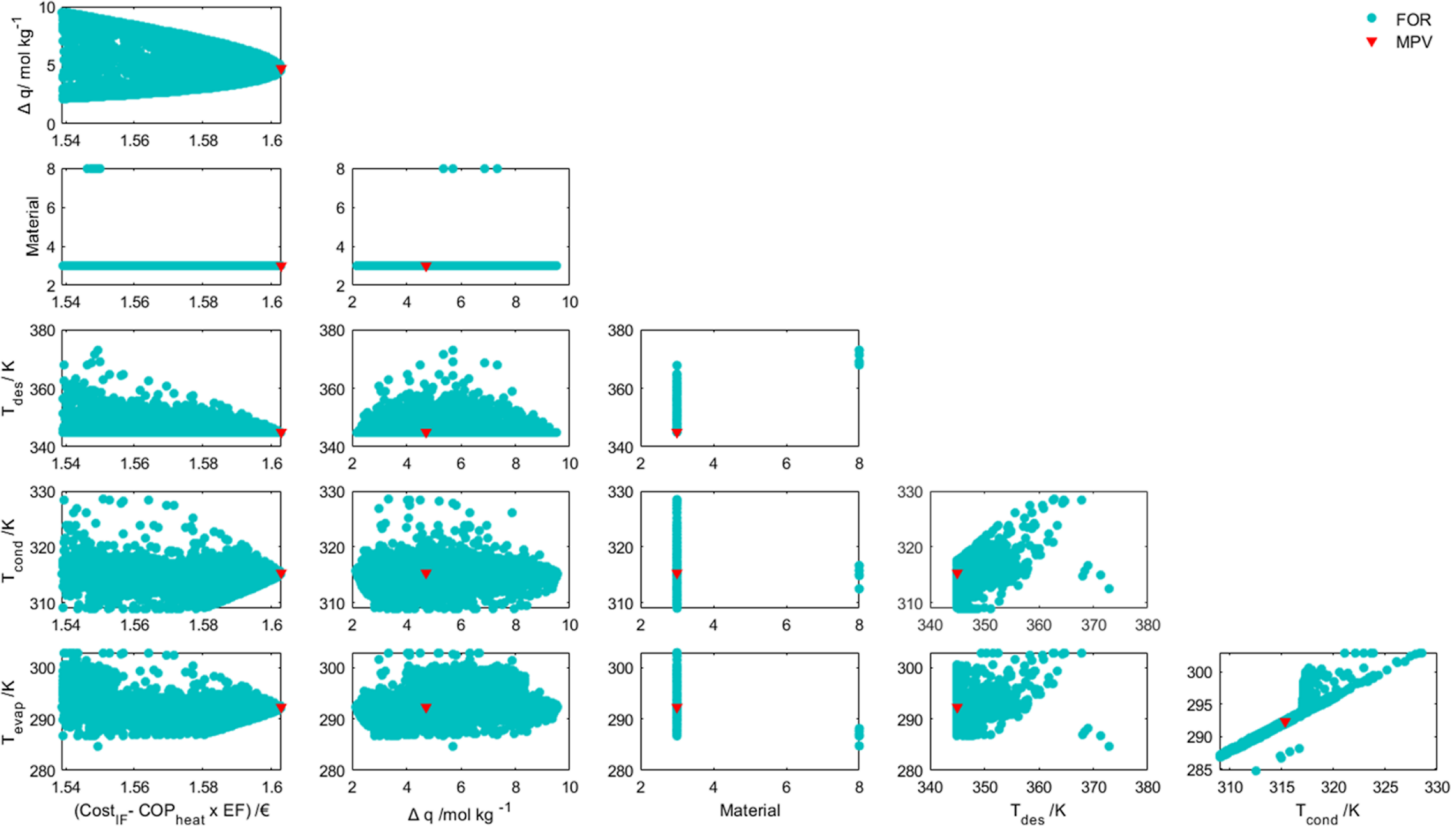
FORs obtained by the Fisher–Snedecor test for *T*_evap_, *T*_cond_, *T*_des_, material, Δ*q*, and (Cost_IF_–COP_heat_ × EF).

The results of the uncertainty assessment are presented in Table 5 based on the FOR. In Figure 7, the FORs are depicted using the absolute value of (Cost_IF_–COP_heat_ × EF).

**Table 5:** Most Probable Values for the Decision Variables and Respective Confidence Intervals for Minimization of (Cost_IF_–COP_heat_ × EF)

decision variable	MPV	CI
*T*evap/K	292.3	[290, 294.6]
*T*cond/K	315.3	[313.3, 317.4]
*T*des/K	345	[345, 347.6]
material	3	[3]; [8]

The materials that best suited the problem remained the same as when the primary objective was to obtain the maximum COP_heat_. The CI for *T*_des_ is more restricted now that cost is a factor in the optimization process and the MPVs for the other two temperatures no longer correspond to the lower bound of the previously defined range.

Based on the conclusion drawn when analyzing the previous case, the points with a Δ*q* lower than 4.7 are the ones with a lower COP_heat_. The data in the FOR for material 8 is above that value, prioritizing the performance of the adsorption-heat pump. The predominance of material 3 is evident, whereas it seemed to be a balance in the last case. Therefore, material 3 has been indicated as adequate for multiple regions of operation, while material 8 has a more restricted application.

When looking at the variation of the adsorbed amount and the OF with the temperatures, the data are dispersed over the high- and low-performance zones. This dispersion was also present in Figure 6. It is then possible to conclude that no single temperature has a predominant influence on the operation of the heat pumps. Instead, the different combination of those three factors is the key to switching between a higher performance and a lower cost function.

This is reinforced by analyzing the plots that relate the three temperatures. *T*_cond_ and *T*_evap_ show an almost-linear dependency, probably due to the relation of these two temperatures with the pressure limits and the enthalpy of vaporization in the condenser. The higher *T*_cond_ is, the lower *q*_max_ is going to be unless *T*_evap_ rises (to raise the pressure at which the adsorption takes place and therefore rising *q*_max_). With the higher *T*_cond_, the enthalpy of vaporization in the condenser becomes lower and less energy is produced by the heat pump.

*T*_des_ seems to be a conflicting temperature. On the one hand, the lower difference between *T*_des_ and *T*_cond_ allows for a lower heat supply requirement for the heat pump. On the other hand, this lower difference causes an inefficient desorption process, especially taking into consideration that it occurs at a higher pressure than adsorption and that pressure is defined precisely by *T*_cond_. This relation is evident in the plots, where the higher values of the desorption temperature only appear for higher values of the adsorption and evaporator temperatures.

In addition, the intertwining between the temperatures also depends on the shape of the isotherms for each material, so the variation in the adsorbed amount can be evaluated for each case. The isosteric heat of adsorption is also an important parameter that influences the energetic cost of desorption.

**Figure 8:**
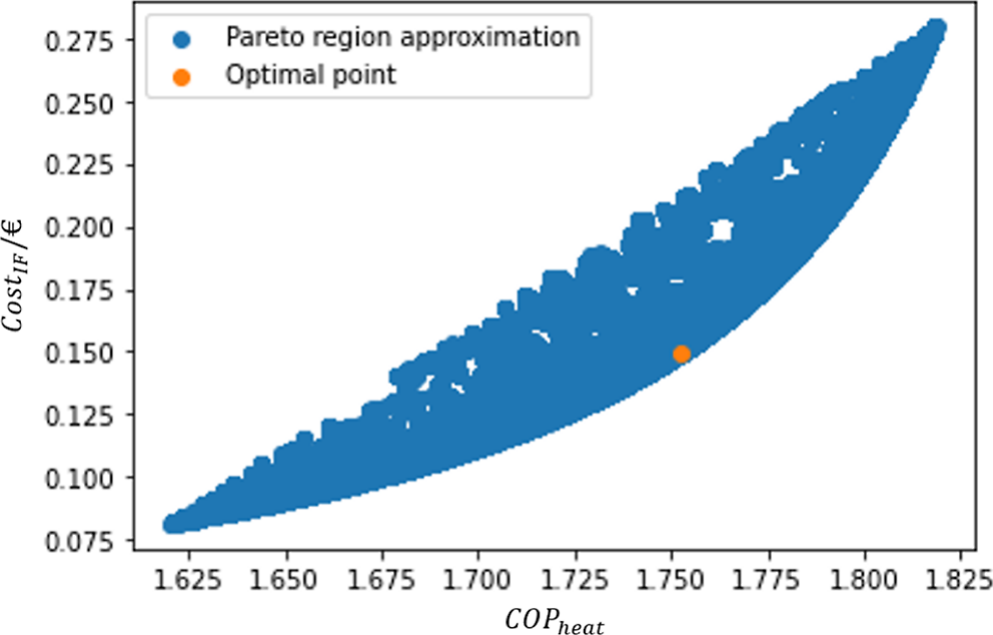
Pareto Region approximation.

With the points in the confidence region, it is possible to calculate the individual values of the OFs and draw an approximation of a limited Pareto Region, as pictured in Figure 8.

The optimal points calculated range between 1.62 and 1.82 for COP_heat_ and 0.08 € to 0.28 € for Cost_IF_, approximately. It is possible to confirm that the objectives are evaluated based on a trade-off deal: to increase the heat pump performance, it is required to increase the cost of the process, while a low cost implies a close-to-zero heat production.

Although this rough design of the Pareto Region is not able to predict the complete evolution of the system within the ranges defined (for example, the point for the maximization of COP_heat_ does not belong in the presented intervals), it seems like a fair estimation for an intermediate behavior zone.

### 3.3 Multi-Objective Optimization

**Figure 9:**
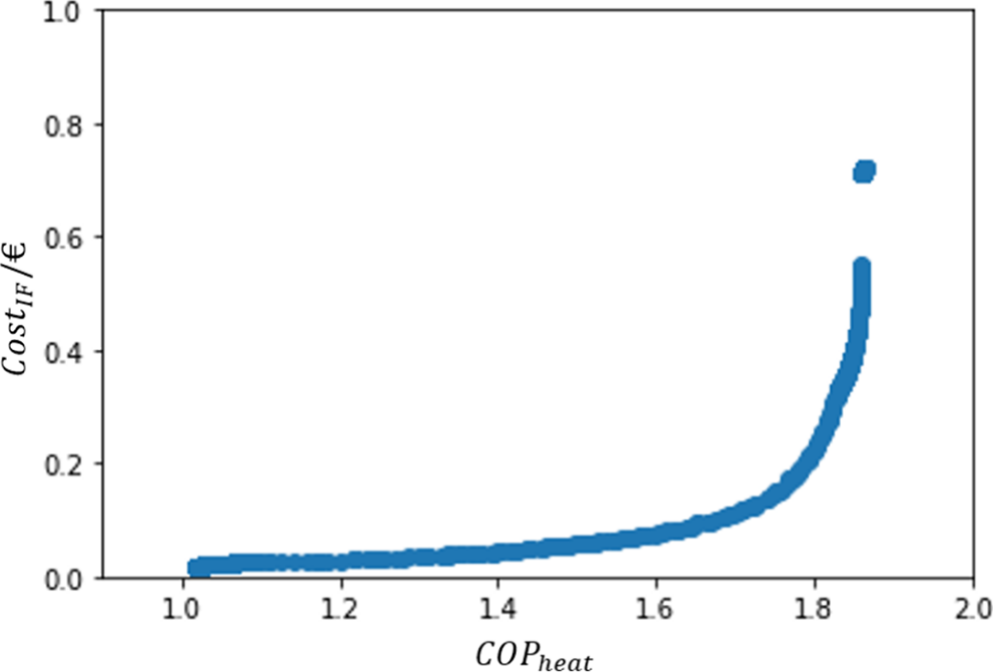
Pareto Front.

The previous section dealt with single-objective optimization and an approximation to a Pareto Front. In this section, an actual Pareto Front will be computed by multi-objective optimization. Hence, it was possible to build a trustworthy Pareto Front, as presented in Figure 9.

It can be observed that the Cost_IF_ values vary between 0.02 and 0.72 €, while the COP_heat_ presented a variation between 1.02 and 1.86.

In addition, it is possible to see a gap within the points of the Pareto Front. This is likely due to the almost vertical development of the front in that area (with no significant increase on COP_heat_ but a rise of circa 0.1 on Cost_IF_), leading to the non-dominance of such points. The upper branch of the curve presented a slightly higher performance and was therefore considered dominant. Another possibility is that there were no feasible points in that region.

**Figure 10:**
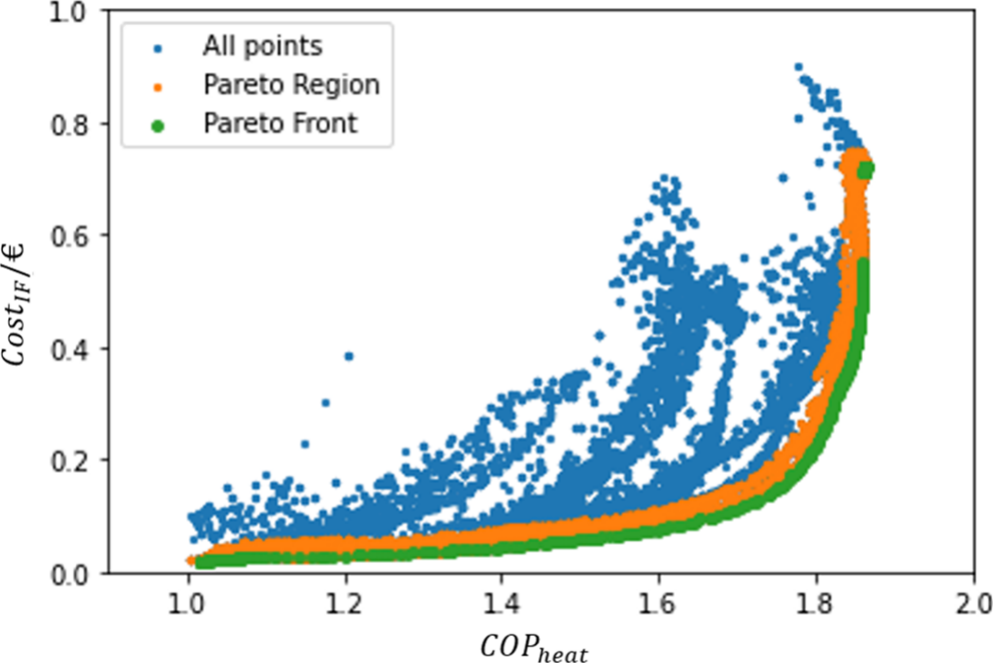
Comparison between all valid data produced by the PSO algorithm, the Pareto Region, and the Pareto Front.

The first hypothesis was confirmed by drawing the Pareto Region according to the Fisher–Snedecor test, which is the orange curve shown in Figure 10. The test was performed with a α = 0.9999, resulting in the inclusion of the *y*_*i*_ values that differed by less than 0.03 (absolute value) from the *y*_best_ points presented in the Pareto curve.

This certifies the importance of drawing the FOR because possible solutions would not be detected otherwise. Furthermore, it can be seen in Figure 10 that the test resulted in a suitable screening of the overall data available. Therefore, all the points in the new Pareto Region, the orange area, can be statistically considered as optimal as those in the Pareto Front.

**Figure 11:**
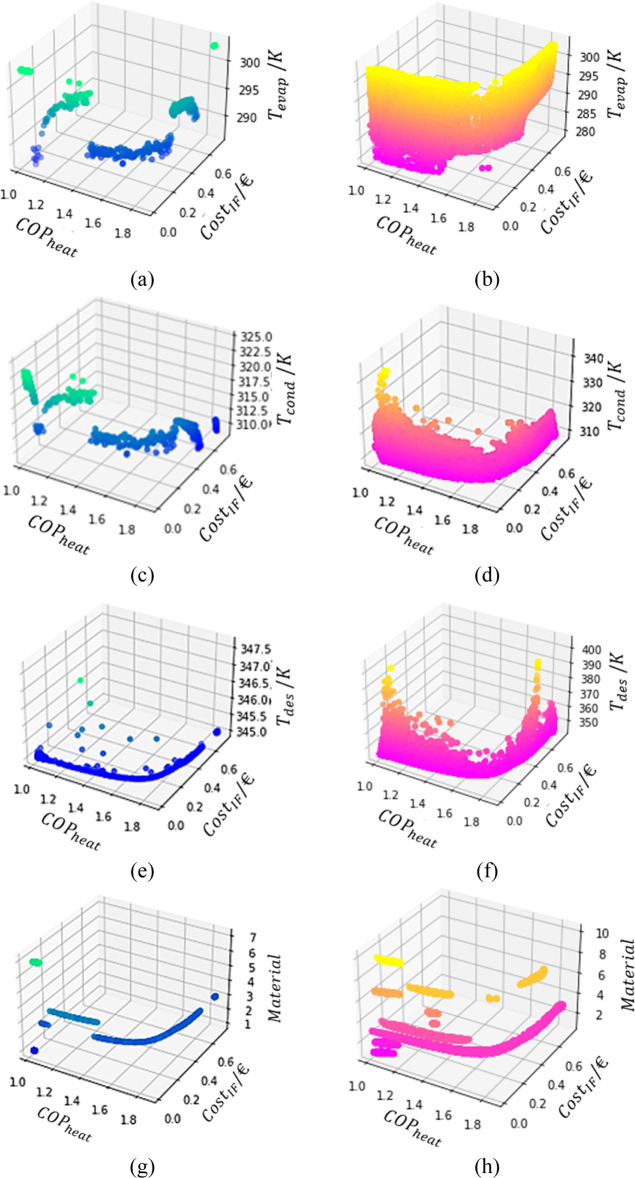
Pareto Fronts (a,c,e,h) and Pareto Regions (b,d,f,h) in functions *T*_evap_ (a,b), *T*_cond_ (c,d), *T*_des_ (e,f), and the adsorbent material (g,h).

For a comparison, the Pareto Front and Pareto Region can be plotted to see the difference in the optimal feasible region for each decision variable, as illustrated in Figure 11.

As can be seen from the comparison of the two Pareto for each variable, the ranges admissible for each variable are vastly expanded with a considerably small variation of the OFs.

The *T*_evap_ shows a significant difference between the two zones. A curious fact is that for lower performances, the full range of the variable can be employed but as the values of COP_heat_ increase, the range becomes smaller, tendentiously to higher values of *T*_evap_.

Similar behavior can be observed for *T*_cond_ = *T*_ads_, where the lower temperatures lead to higher performances.

The variable *T*_des_, which in the Pareto Front was mostly constant at 345 K, shows two peaks in temperature achieving almost the 400 K value in the Pareto Region. This is an odd event as for higher *T*_des_, the expected result would be a higher cost since more heat would be spent heating the adsorbent bed. This might be connected to the adsorbent materials since the expansion of the admissible results for the OFs resulted in the opportunity to apply different materials that did not appear to be suitable before.

**Figure 12:**
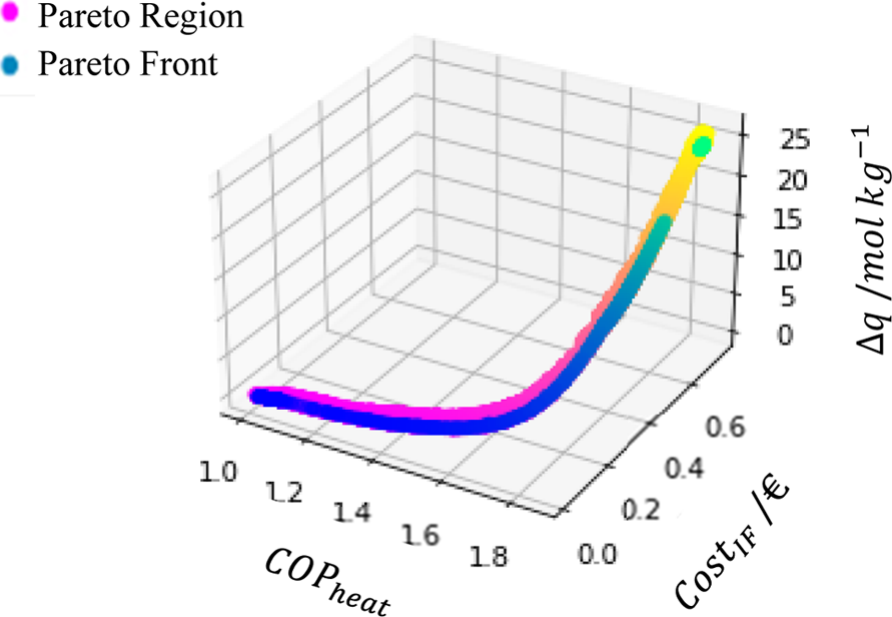
Relation between Δ*q* with the Pareto Front and Pareto Region.

To find an explanation for the value of 400 K in the zone of low cost, the Δ*q* obtained with the particles within the FOR was plotted against the cost, the COP_heat_, and the Cost_IF_ as visualized in Figure 12.

For the zone of worst performance and lower cost, the Δ*q* is nearly 0, and as the difference between the maximum and the minimum adsorbed amounts rises, both cost and the coefficient of performance increase.

The variation of COP_heat_ can be explained by considering that the main contribution to this value is made by the heat released during the water condensation, as described in the literature since *Q*_heat_ + *Q*_des_ very often acquires a value similar to *Q*_cool_ + *Q*_ads_.^4^ If Δ*q* = 0, no water will be condensed, explaining the COP_heat_ value near 1.

When it comes to cost, when Δ*q* = 0, the heat required is practically for increasing the adsorbent and adsorbate temperatures. As predicted, the heat produced is equal to that provided, and there is no point in using the adsorption heat pump.

**Figure 13:**
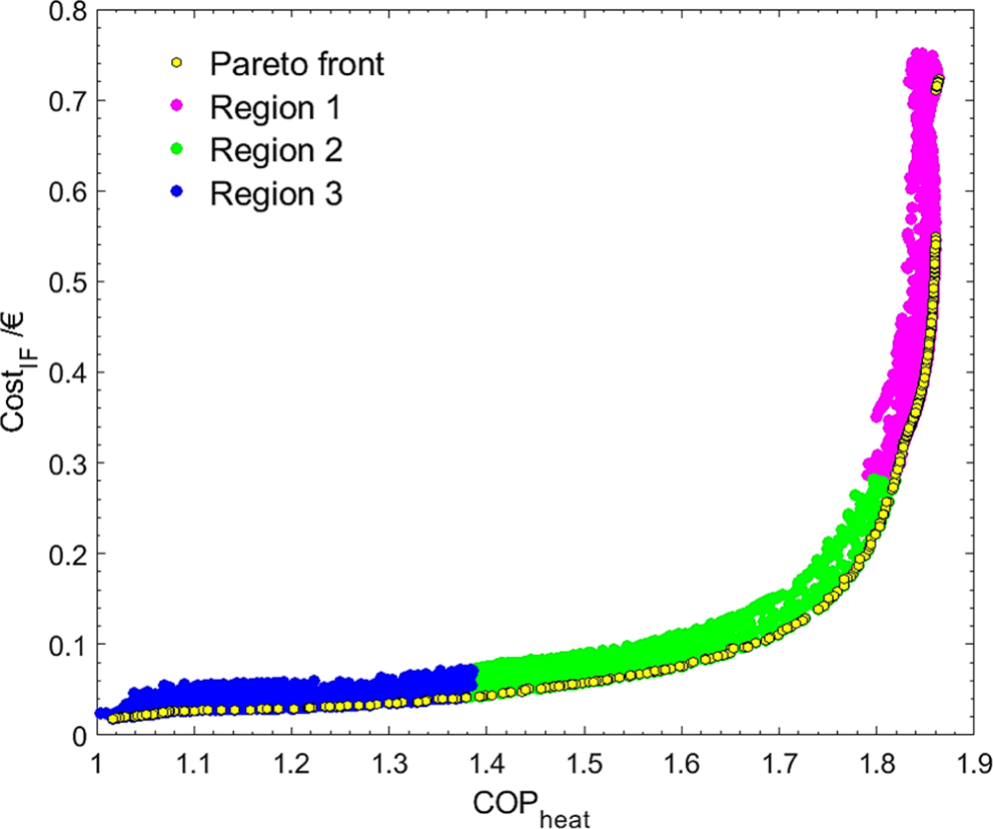
Clusterization of the data within the Pareto Region.

**Figure 14:**
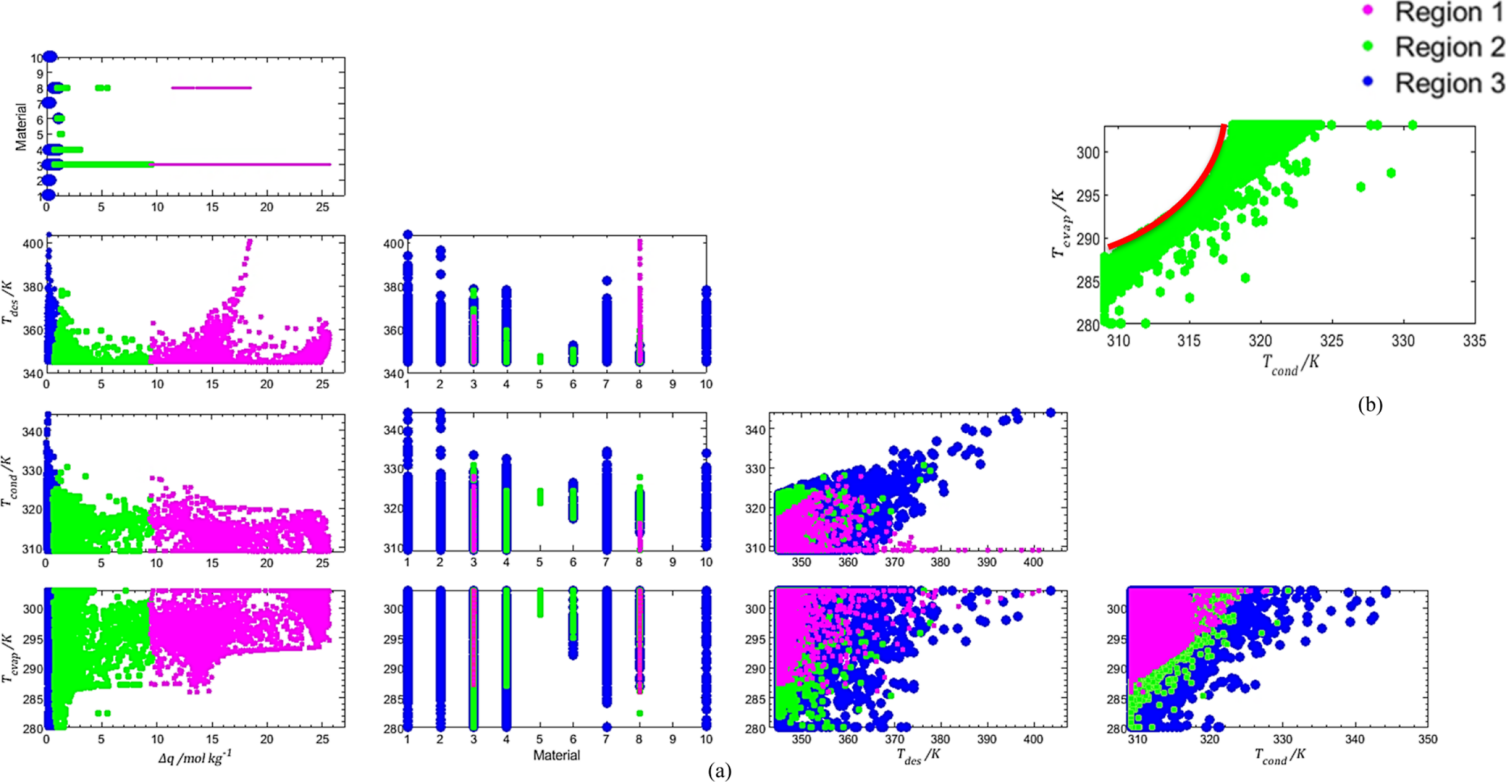
FORs obtained by the Fisher–Snedecor test for *T*_evap_, *T*_cond_, *T*_des_, Material, and Δ*q* (a) and the detail of *T*_evap_ vs *T*_cond_ for Region 2 (b).

The Pareto Region data were grouped into clusters to understand each OF’s predominance better. The limits of the OFs in each cluster and the number of points in each region are presented in Table 6. In Figure 13, the representations of the Pareto Front and the different FORs are presented. The distribution of the decision variables in each cluster is pictured in Figure 14. The Δ*q* was also plotted to provide a better idea of the values of COP_heat_ and Cost_IF_ resulting from the different combinations of the decision variables.

**Table 6:** Variation of COP_heat_ and Cost_IF_ and Number of Points within Each Region

region	COP_heat_	Cost_IF_	number of points
1	[1.02, 1.39]	[0.02, 0.07]	22046
2	[1.39, 1.82]	[0.07, 0.27]	14025
3	[1.82, 1.86]	[0.27, 0.75]	42578

Regions 1 and 3 present more points than Region 2, meaning the convergence was easier in zones where one of the OFs was dominant, opposing the optimal calculation during the single optimization.

It can be seen by the plots that temperatures generally overlapped and *T*_cond_ and *T*_des_ tend to lower values while *T*_evap_ rises.

The relation between *T*_cond_ and *T*_evap_ for Region 2 is presented in detail in Figure 14b. The temperature of the evaporator is always about 20 K lower than the temperature of the condenser, resembling the tendency observed before.

This limitation does not extend to the other two zones. In zone 3, the Δ*q* is low, so the temperatures can range freely. In zone 1, the adsorbed amount is significantly higher, ranging from 10 to 27 mol kg^–1^, despite the absence of the temperature limitation. In fact, the plot of *T*_cond_ vs *T*_evap_ for zone 3 is almost complementary to the one of zone 2, indicating that above the limit drawn for zone 2, the values of COP_heat_ and Cost_IF_ reach their maximum values.

The main factor seems to be the different materials at play.

Both MIL-100 (Fe) (material 3) and MIL-125_NH_2_ (Ti) (material 8) showed high capacities of adsorption at low pressures and relatively low isosteric heats of adsorption, leading to a greater performance under the most variable conditions. These two materials are present in Regions 1, 2, and 3, so they would be a good option for applications where the conditions could be regulated to choose a greater amount of heat produced or a lower-cost operation.

Nevertheless, MIL-100 (Fe) was the material with the most consistent performance, as seen by the continuous evolution of Δ*q* across the three different zones. Furthermore, the high variation in the adsorbed amount can contribute to integrating adsorption based-heat pumps into water recovery systems,^46^ but further studies would be required to confirm this hypothesis.

MIL-160 (Al) (material 6), Al-FUM (material 4), and AQSOA FAM-Z02 (material 5) were also favorable materials for a midterm operation of the heat pumps. The MOFs can perform in a larger range of temperatures. However, the AQSOA FAM-Z02 was the only material to be exclusively considered for the intermediate region. This is probably due to the sharp isotherm, which makes desorption harder, and the high (−Δ*H*_ads_) value that this material presents increasing the cost of the desorption operation. In fact, the range for the desorption temperature is the narrowest of all the evaluated materials.

The other materials showed low performance and poor adsorption/desorption capacity under the conditions studied. Most of them also possess a high isosteric heat of adsorption, which might contribute to the lower performance displayed during the optimization.

The uncertainty assessment results for each region are displayed in Table 7.

**Table 7:** Most Probable Values and Confidence Intervals for the Decision Variables in Each Region

	region 1		region 2		region 3	
decision variable	MPV	CI	MPV	CI	MPV	CI
*T*evap/K	297.5	[290.0, 303.0]	297.2	[286.0, 303.0]	298.4	[288.1, 303.0]
*T*cond/K	313.0	[309.0, 318.8]	317.8	[310.3, 325.1]	317.1	[309.4, 324.7]
*T*des/K	346.6	[345.0, 355.6]	345.9	[345.0, 350.95]	346.6	[345.0, 355.2]
material	3	[3]; [8]	3	[3,6]; [8]	7	[1,4]; [6,8]; [10]

The *T*_evap_ and *T*_des_ values are very similar between regions even though the confidence interval for *T*_des_ in Region 2 is slightly more restricted than in the other regions. The most well-known difference appears to be the adsorption temperature, which is higher in the lower cost zones. These values reflect the overlapping in the plots in Figure 12.

The MPVs were used to calculate the corresponding values of COP_heat_ and Cost_IF_ that can be seen in Table 8. Despite the slight difference in temperatures between Region 1 and Region 2, it translates into a significant variation in cost and performance for the same material while agreeing to each cluster’s framing values.

**Table 8:** Values of COP_heat_ and Cost_IF_ Obtained with the MPVs for Each Region

objective function	region 1	region 2	region 3
COP_heat_	1.85	1.79	1.08
Cost_IF_	0.69	0.26	0.03

The fact that a slight variation in the temperature values leads to such a difference in the adsorption-heat pump performance emphasizes the importance of mapping the performance regions for each material.

A form of doing this is through analyzing Figure 14, following the paths demonstrated in Figures SI.24 and SI.25 in the Supporting Information. With that line of thinking, it is possible to evaluate the odd point that appeared during the analysis of Figure 11. The point in zone 3 that presents a 410 K for desorption temperature corresponds to material 1, *T*_cond_ = 344 K and *T*_evap_ = 303 K, leading to a COP_heat_ of 1.12 and a Cost_IF_ of 0.06 €, with a Δ*q* of 0.33 mol kg^–1^. The point for Region 1 with the same desorption temperature is obtained by using material 8, *T*_cond_ = 309 K and *T*_evap_ = 303 K, leading to a COP_heat_ of 1.83 and a Cost_IF_ of 0.57 €, with a Δ*q* of 18.5 mol kg^–1^. The low Δ*q* for the point in Region 1 justifies the low COP_heat_ and the low cost.

**Figure 15:**
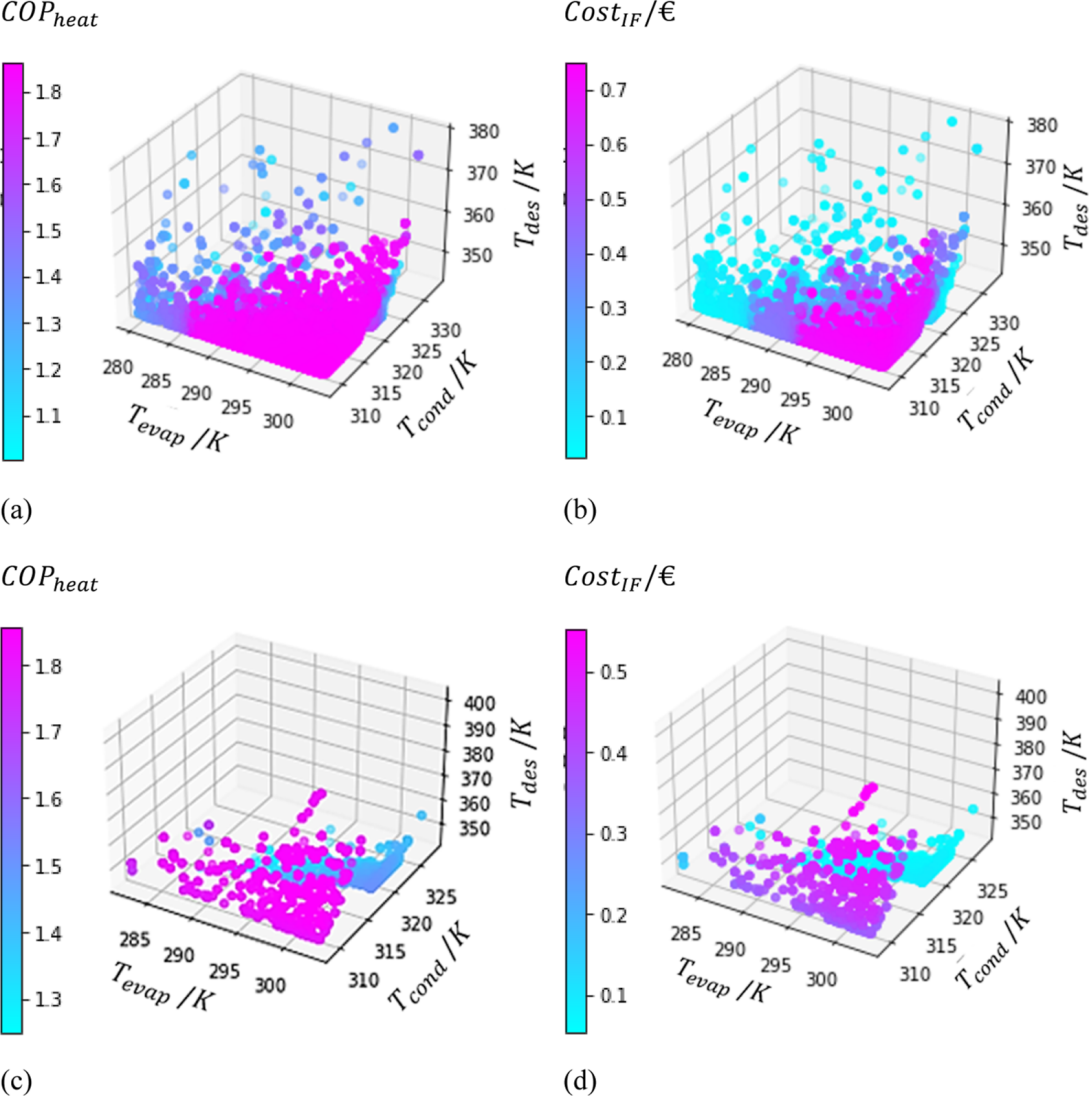
Temperature maps for materials 3 [MIL-100 (Fe)] (a,b) and 8 [MIL-125_NH_2_ (Ti)] (c,d) for COP_heat_ (a,c) and the Cost_IF_ (b,d).

However, this logic is difficult to apply when the temperature plots are overlapped. An alternative is to plot a 3D graph correlating the three temperatures and the OFs with the particles belonging to the Pareto Region for each material individually, as illustrated for MIL-100 (Fe) and MIL-125_NH_2_ (Ti) in Figure 15.

The difference in the number of points for each material is well known since only the optimal points were used. MIL-100 (Fe) allows for a more considerable temperature variability, which can be useful to control the heat transfer phenomenon when using the power supplied by the heat pump. Namely, operating at higher temperatures of the condenser can be more effective in heating since there will be a bigger gradient of temperatures.

However, the optimal performance point for MIL-125_NH_2_ (Ti) leads to a lower cost when compared to MIL-100 (Fe). Furthermore, MIL-125_NH_2_ (Ti) seems to perform better even with lower *T*_evap_. Therefore, these plots confirm the importance of evaluating multiple temperature combinations because different materials can be suited for different zones of operation. These plots are also of great value for control operations since they map the temperature ranges in which the performance and the cost remain similar to the one intended.

### 3.4 Comparison between Single- and Multi-Objective Optimizations

It was interesting to compare the results of the optimization. Both values obtained in cluster Regions 1 and 2 were very similar to those obtained when maximizing COP_heat_ or having a single-objective approach to the multi-optimization problem. Nevertheless, the analysis of optimal points provided by the single-objective method was limited.

Regarding the MPV and the IC, the multi-objective approach led to wider intervals than the single-objective one. One of the main differences was the MPV values for Region 1 in multi-optimization (corresponding to maximum values of COP_heat_), which did not hit the side constraint values as in the single-objective, most likely due to the cost limitation. In addition, the MPV for Region 2 led to a higher cost and performance than in the single objective. Hence, most points in that area leaned toward the greatest performance over cost.

This provides evidence that multi-optimization is capable of a more consistent evaluation of the multi-objective problem compared with single-optimization. This conclusion is in line with the knowledge of optimization theory.

**Figure 16:**
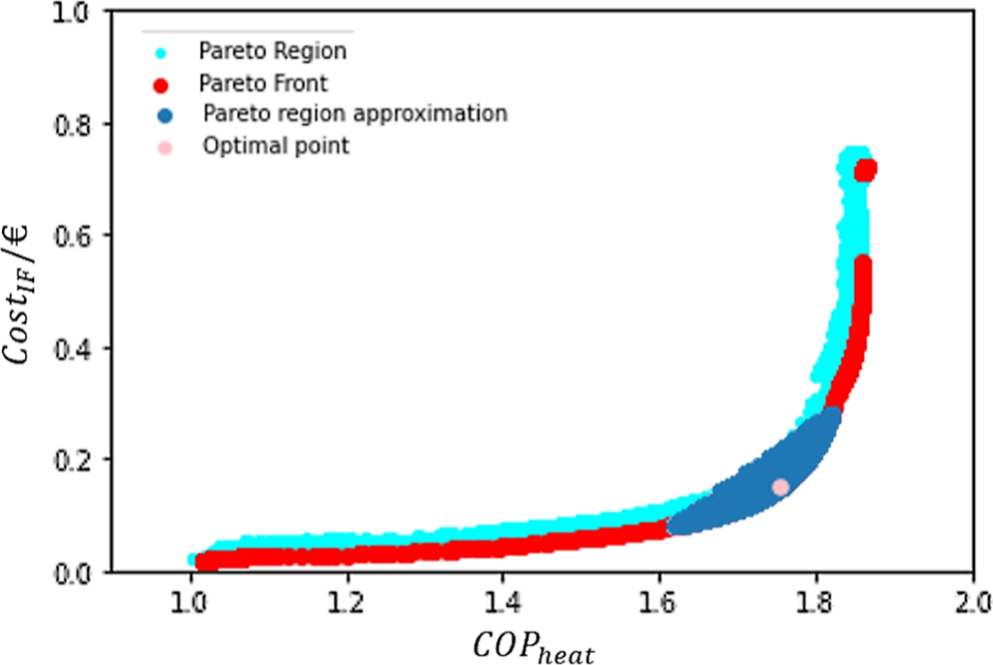
Comparison of optimal regions for the single objective approach and the multi-objective optimization.

This can be perceived more easily when comparing the approximated Pareto Region obtained for the single-objective optimization of (Cost_IF_–COP_heat_ × EF) with the Pareto Region obtained for the multi-objective optimization, as pictured in Figure 16.

The Pareto curve gives a broader perspective of the overall problem. In contrast, the approximation achieved with the single-objective approximation is restricted to the area of compromise between the two regions. The single-objective fails to identify MIL-160 (Al), Al-FUM, or AQSOA FAM-Z02 as possible solutions for the mid-cost operation.

Furthermore, the approximated Pareto Region will consider all the points that achieve a difference within the given confidence level in the Fisher–Snedecor test. Some points of that region might not deliver a good performance or be of a lower cost and still have a difference between those values that makes them belong to the FOR.

Therefore, a single-objective is an excellent tool for finding higher-performance zones without limitations. When the objective is to find a compromise to different objectives, multi-objective optimization is the more indicated tool for the general overview of the problem.

### 3.5 Decision-Making on Material and Operation Modes

The optimization procedure presented until this point produced a multiplicity of results relevant to decision-making.

As can be seen in Figures 6 and 7, a vast combination of variables can be used to achieve objectives within less than 4% variation from the MPV for the optima. This is the same as saying that the single-objective approach allows for obtaining the parameters’ interval and combinations that lead to a small-to-none variation in the practical application of adsorption heat pumps.

A similar line of thought can be applied to the multi-objective optimization, with the choice being oriented by the desired mode of operation. In the most rigorous winter, the cost of operation might not be the most concerning matter when one is trying to obtain heat, Region 1 of Figures 13 and 14 being preferred. If one is preferring to cut on energetic expenses, Region 3 provides more fitted arrangements to achieve heat production within a low-cost operation. When neither of the objectives is a priority, the points over the Region 2 will be adequate.

After choosing the adsorbent employed in the unit, it is essential to attend to graphics as Figure 15 to evaluate the ranges of operating temperatures for the desired operation.

At last, it is also relevant to confirm that these results present the basis for decision-making in material screening. Other factors could sway the decision between the optimal points retrieved, such as the adsorbent cost or the resulting volume of the operating unit if one considers domestic applications.

## 4 Conclusions

The novel material screening strategy successfully chose adsorbents for maximizing performance and/or minimizing operation costs. The present work contributed to new insightful uses of PSO algorithms in mixed-integer problems, optimizing both the choice of adsorbent and the operating temperatures.

The application of the Fisher–Snedecor statistical test was crucial for the broadening of the optima results to expand the possible optima combinations. This extension represented the consideration of other points within a 4% decrease in the value of the objectives when compared to the optima point in each case. In practical applications, all those points are statistically identical, with no prejudice in operation.

A significant remark is a similar performance obtained for MIL-100 (Fe) and MIL-125_NH_2_ (Ti) in both single-objective approaches, which would likely not be perceived with the traditional material screening methods. Besides this, the expansion of viable optimal operating conditions was indisputable.

Regarding the multi-objective approach, the Pareto Region clusterization was a key step toward better decision-making based on which objective is more preponderant at the moment. It allowed the evaluation of which zone was more appropriate for each material or the temperature ranges that promoted the switch between operation modes. The arrangement of the temperature data for each material provided a powerful tool to evaluate the impact of temperature variation on the unit’s control.

Furthermore, this trailblazing tool brings a lot of advantages to material screening in adsorption heat pumps. For starters, the time it would take to evaluate all the different temperatures largely surpasses the computational time spent in the implementation of the algorithm proposed here. Moreover, the methodology can easily be expanded to novel materials and temperature ranges. Finally, expanding the optimal results allowed for a better understanding of the process and the respective control operations.

Nevertheless, this approach reveals some limitations. The more points included in the FOR, the more computational difficulties will be presented in the clusterization procedure. In addition to this, some other variables and objectives can contribute to the decision-making procedure but were not included in this study, such as the adsorbents’ price or the volume of the unit.

The presented framework can be expanded in future works. An interesting point would be the expansion of this framework to cooling applications. Another interesting proposal would be to change the model used to describe the adsorption heat pump operation to contemplate a dynamic component and evaluate the trade-off between the solutions’ accuracy and the inevitable increase in computational time.

## Supporting Information Available

Additional information about the model used in the simulation, adsorbent data, and the strategy for temperature mapping (PDF)

## 5. Notation

*a*^*v*^: initialization factor of velocity, [−]
*C*_0_: acceleration coefficient [−]
*C*_0,*f*_: final value of *C*_0_ [−]
*C*_0,*i*_: initial value of *C*_0_ [−]
*C*_1_: acceleration coefficient [−]
*C*_2_: acceleration coefficient [−]
CF: coverage factor [−]
COP_heat_: coefficient of performance for heating applications [−]
Cost: cost of an adsorption heat pump operating cycle [€]
Cost_GN_: cost of natural gas [€ GJ^–1^]
Cost_IF_: cost with impact factor [€]
EF: equalization factor [€]
*G*_best_: vector of the best positions for global swarm [−]
IF: impact factor [−]
*k*: current iteration [−]
LF: loss factor [−]
*N*: number of points [−]
*N*_DV_: number of decision variables [−]
*N*_exp_: number of experiments [−]
maximum number of iterations [−]
*N*_OF_: number of objective functions [−]
maximum number of particles [−]
*P*: pressure [bar]
*P*_best_: vector of the best positions for each particle [−]
*P*_cond_: condensation pressure [bar]
*P*_evap_: evaporation pressure [bar]
*q*: adsorbed amount [mol kg^–1^]
*q*_max_: maximum adsorbed amount [mol kg^–1^]
*q*_min_: minimum adsorbed amount [mol kg^–1^]
*Q*_ads_: heat of isobaric adsorption [J]
*Q*_cond_: heat of condensation [J]
*Q*_cool_: heat of isosteric cooling [J]
*Q*_des_: heat of desorption [J]
*Q*_evap_: heat of evaporation [J]
*Q*_heat_: heat consumed during the isosteric heat phase [J]
*r*: random number [−]
*T*: temperature [K]
*T*_2_: intermediate temperature [K]
*T*_ads_: temperature of adsorption [K]
*T*_4_: intermediate temperature [K]
*T*_cond_: condensation temperature [K]
*T*_des_: temperature of desorption [K]
*T*_evap_: evaporation temperature [K]
*U*,: uncertainty (standard deviation within the feasible operating region data) [−]
*v*: velocity of the particles [−]
*V*: matrix containing the velocities of all particles during the PSO implementation [−]
*v*_*i*,*n*,*k*_: velocity for each particle in each decision variable for each interaction [−]
*w*: maximum violation rate [−]
*w*_L_: maximum violation rate for lower side constraints [−]
*w*_U_: maximum violation rate for upper side constraints [−]
*x*: particle position [−]
*X*: matrix containing the positions of all particles [−]
*x*_*i*,*n*,*k*_*: candidate position [−]
*x*_*i*,*n*,*k*_: accepted position [−]
*x*_*n*,lower_: lower side constraints of decision variables [−]
guide position within global best [−]
guide position for each particle [−]
*x*_*n*,upper_: upper side constraints of decision variables [−]
*y*(*x*): objective function vector for each set of positions [−]
*Y*_best_: matrix of objective function values for a particle belonging to *G*_best_ [−]
*Y*: matrix of all the objective function values produced by a particle during the PSO optimization [−]

## Greek letters

α: level of confidence [−]
(−Δ*H*_ads_): isosteric heat of adsorption [J mol^–1^]
Δ*q*: difference between *q*_min_ and *q*_max_ [mol kg^–1^]

## Indexes

*I*: particle
*N*,: decision variable
*k*: iteration

## List of Acronyms

CI: confidence intervals
CSPSO: constrained sliding particle swarm optimization
FOR: feasible operating region
LHS_MDU: latin hypercube sampling with multi-dimensional uniformity
MOF: metal–organic framework
MPV: most probable value
OF: objective function
PSO: particle swarm optimization
RAM: random access memory
