# Supplementary material for: Metaheuristic Framework for Material Screening and Operating Optimization of Adsorption-Based Heat Pumps
Source: ACS Omega. 2023 May 23;8(22):19874–91. doi: 10.1021/acsomega.3c01797 (PMC10249114; doi:10.1021/acsomega.3c01797)
Supplement: Supplementary file 1 — ao3c01797_si_001.pdf [file ao3c01797_si_001.pdf]

Metaheuristic framework for material screening and operating optimization of adsorption-based heat pumps

Beatriz C. Silva [a, b]*, Carine Rebello [c], Alírio E. Rodrigues [a, b], Ana M. Ribeiro [a, b], Alexandre F. P. Ferreira [a, b] and Idelfonso B. R. Nogueira [d,*]

a. LSRE-LCM - Laboratory of Separation and Reaction Engineering – Laboratory of Catalysis and Materials, Faculty of Engineering, University of Porto, Rua Dr. Roberto Frias, 4200-465 Porto, Portugal

b. ALiCE - Associate Laboratory in Chemical Engineering, Faculty of Engineering, University of Porto, Rua Dr. Roberto Frias, 4200-465 Porto, Portugal

c. Chemical Engineering Department, Polytechnic School Federal University of Bahia, Salvador 40210-630, Brazil

d. Chemical Engineering Department, Norwegian University of Science and Technology, Sem Sælandsvei 4, Kjemiblokk 5, Trondheim, Norway

* Correspondence: up201707213@up.pt; idelfonso.b.d.r.nogueira@ntnu.no

Thermodynamic model

In this model, the heat transferred in isosteric heating (Q_{heat}), isobaric desorption (Q_{des}), isosteric cooling (Q_{cool}), and isobaric adsorption (Q_{ads}), as well as in condensation (Q_{cond}) and evaporation (Q_{evap}) were calculated by the equations SI.1 to SI.6.

$$Q_{heat} = \int_{T_{ads}}^{T_2} m_{ads}\left(c_{p,ads}(T) + q_{max}\, c_{p,water}(T)\right) dT \quad \text{(SI.1)}$$

$$Q_{des} = \int_{T_2}^{T_{des}} m_{ads}\left(c_{p,ads}(T) + q(T)\, c_{p,water}(T)\right) dT + \int_{q_{max}}^{q_{min}} m_{ads}\,(-\Delta H_{ads})(q)\, dq \quad \text{(SI.2)}$$

$$Q_{cool} = \int_{T_{des}}^{T_4} m_{ads}\left(c_{p,ads}(T) + q_{min} c_{p,water}(T)\right) dT \quad \text{(SI.3)}$$

$$Q_{ads} = \int_{T_4}^{T_{ads}} m_{ads}\left(c_{p,ads}(T) + q(T)\, c_{p,water}(T)\right) dT + \int_{q_{min}}^{q_{max}} m_{ads}\,(-\Delta H_{ads})(q)\, dq \quad \text{(SI.4)}$$

$$Q_{evap} = m_{ads}\, \Delta q\, \Delta H_v + \int_{T_{cond}}^{T_{evap}} m_{ads}\, \Delta q\, c_{p,water}(T)\, dT \quad \text{(SI.5)}$$

$$Q_{cond} = m_{ads}\, \Delta q\, \Delta H_v \qquad \text{(SI.6)}$$

Where m_{ads} it the mass of adsorbent, $c_{p,ads}$ is the specific heat capacity of the adsorbent, q_{max} and q_{min} are the maximum and minimum adsorbed quantities during the cyclic operation, with $\Delta q = q_{max} - q_{min}$, $c_{p,water}$ is the water specific heating capacity, $(-\Delta H_{ads})$ is the isosteric heat of adsorption, and ΔH_v is the vaporization enthalpy of water.

Unlike some thermodynamic models presented in the literature [1–4], the mass of inerts (the mass of metal tubes in the adsorbent heat exchanger, for example) was disregarded since this simulation process is not based on experimental data and, therefore, there would be no valid foundation in the value attributed to the said parameter.

Furthermore, every term of Equations SI.1-SI.6 depends on the adsorbent mass. So, a value of 1 kg was assumed because changing it would be equivalent to a simple multiplication of the obtained value by the mass of the adsorbent.

Regarding the temperatures of the system, T_{ads} was considered equal to T_{cond} [5]. Besides that, the intermediate temperatures (T_2 and T_4) can be calculated based on the remaining temperatures with Equations SI.7 and SI.8 [6–8].

$$T_2 = \frac{T_{cond}^2}{T_{evap}} \qquad \text{(SI.7)}$$

$$T_4 = \frac{T_{evap}\ T_{des}}{T_{cond}} \qquad \text{(SI.8)}$$

The value of water's heat capacity in J mol^{-1} K^{-1} was assumed to depend on the system temperature according to Equation SI.9 [9].

$$c_{p,\,water}(t) = \left(A + B\,t + C\,t^2 + D\,t^3 + \frac{E}{t^2}\right) \qquad \text{(SI.9)}$$

With $t = \frac{T}{1000}$, $A = -203.6060, B = 1523.290, C = -3196.413, D = 2474.455,$ and $E = 3.855326$ in the corresponding units.

The heat of vaporization of water in J mol^{-1} is calculated by Equation SI.10 [8].

$$\Delta H_v = (2502 - 2.51\,(T - 273)) \times 18.02 \quad \text{(SI.10)}$$

The limits for the adsorbed water amount are determined by $q_{max} = q\ (T_{ads}, P_{evap})$ and $q_{min} = q\ (T_{des}, P_{cond})$. The values of P_{cond} and P_{evap} in bar are calculated through the Antoine Equation (Equation SI.11) with the values of T_{cond} and T_{evap} in Kelvin [10].

$$P = 10^{A - \frac{B}{C+T}} \quad \text{(SI.11)}$$

With $A = 4.6543$, $B = 1435.264$, and $C = -64.848$ in the corresponding units for temperatures and between 255.9 K and 375 K.

The parameters q,ΔH_{ads} and $c_{p,ads}$ depend on the adsorbent material and are presented in the following section.

The integration of Equations SI.1-SI.6 was solved by resorting to Simpson's 3/8 rule.

Adsorbents data

Adsorption isotherms

For MIL-100 (Fe), MIL-125_NH$_2$ (Ti), MIL-160 (Al), CAU-10, and Al-FUM, the data of water adsorption and the isotherm model parameters were retrieved from previous works developed in LRSE-LCM [11–14], with the adsorbent being supplied by KRICT (Korea Research Institute of Chemical Technology).

The other isotherms were fitted based on the experimental data collected from scientific articles, either from a direct collection of the values of the adsorbed quantities or the graphic reading using the ScanIt software. The data was collected from different sources and compared to choose the most consensual dataset with the larger amplitude in terms of temperature and pressure. That dataset was then fitted to the Ising-Langmuir isotherm equation, presented in Equation SI.12 [14].

$$q = q_{sat\,I}\frac{K_0\,P}{(K_0\,P + w_I^2)} + q_{sat\,L}\frac{K_L\,P}{(1 + K_L\,P)} \tag{SI.12}$$

With

$$w_I = \frac{1}{2}\left(1 - K_I\,P + \sqrt{(1 - K_I\,P)^2 + 4K_0\,P}\right) \tag{SI.13}$$

$$K_I = K_{\infty,\,I}e^{\left(\frac{-\Delta H_I}{RT}\right)} \tag{SI.14}$$

$$K_0 = K_{\infty,0}e^{\left(\frac{-\Delta H_0}{RT}\right)} \tag{SI.15}$$

$$K_L = K_{\infty,L}e^{\left(\frac{-\Delta H_L}{RT}\right)} \tag{SI.16}$$

Where q is the total adsorbed amount, $q_{sat\,I}$ is the specific saturation adsorption capacity in Ising isotherm, $q_{sat\,L}$ is the specific saturation adsorption capacity in Langmuir isotherm, and $K_{\infty,\,I}$, $K_{\infty,0}$ and $K_{\infty,L}$ are the equilibrium constants of Ising-Langmuir isotherm, R is the ideal gas constant and the temperature dependence is given by the van't Hoff law.

For the water adsorption isotherm in MIL-100 (Fe), the model used was the Dual-Ising Single-Langmuir (DISL) model, presented in Equation SI.17 [14].

$$q = \sum_{i\,=\,A,B}\left(q_{sat\,I}\frac{K_{o,i}\,P}{(K_{o,i}\,P + w_{I,i}^2)}\right) + q_{sat\,L}\frac{K_L\,P}{(1 + K_L\,P)} \tag{SI.17}$$

Where A and B represent each one of the MIL-100 (Fe) cages, and the parameters are calculated by Equations SI.13- SI.16.

The parameters for the isotherm equation were calculated using the solver from Excel to minimize the absolute error between the estimated adsorbed amount and the corresponding value from the dataset for a specific pressure value.

In the following subsections, the considered water adsorption isotherms for each material are presented, as well as the parameters obtained during the fitting operation. Overall, the adjusted isotherm curves shows a good agreement to the experimental data acquired from the literature.

MIL-100 (Fe)

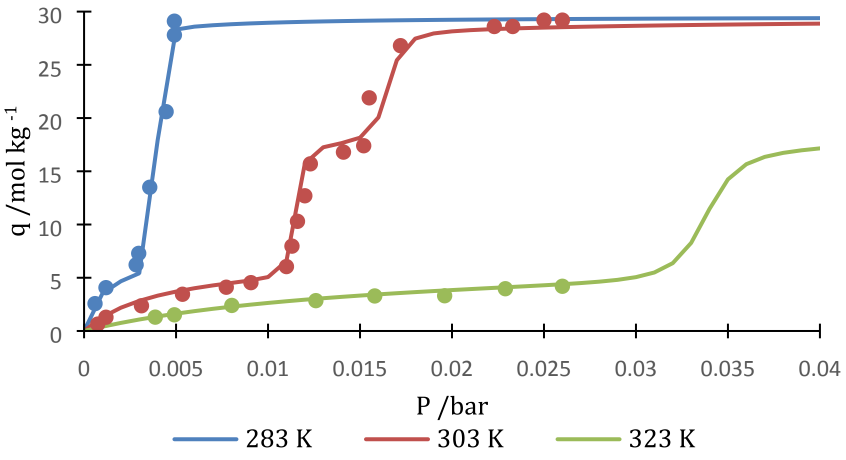

Figure SI. 1. MIL-100 (Fe) dataset fitting to the DISL isotherm.[11,14]

Table SI. 1. DISL parameters for water adsorption in MIL-100 (Fe).[11,14]

Parameter	Value	Parameter	Value
$q_{sat\,L}$ /mol kg^{-1}	6.84	$K_{\infty,\ \mathrm{L}}$ /bar^{-1}	1.15 × 10^{-7}
$q_{sat\,I,1}$ /mol kg^{-1}	12.3	$-\Delta H_{I,1}$ /kJ mol^{-1}	43.9
$q_{sat\,I,2}$ /mol kg^{-1}	10.4	$-\Delta H_{I,2}$ /kJ mol^{-1}	46.8
$K_{\infty,\ I,1}$ /bar^{-1}	2.35 × 10^{-6}	$-\Delta H_{o,1}$ /kJ mol^{-1}	20.5
$K_{\infty,\ I,2}$ /bar^{-1}	5.17 × 10^{-7}	$-\Delta H_{o,2}$ /kJ mol^{-1}	1.33
$K_{\infty,\ \mathrm{o},1}$ /bar^{-1}	1.04 × 10^{-5}	$-\Delta H_L$ /kJ mol^{-1}	54.0
$K_{\infty,\ \mathrm{o},2}$ /bar^{-1}	2.34 × 10^{2}		

MIL-125_NH$_2$ (Ti)

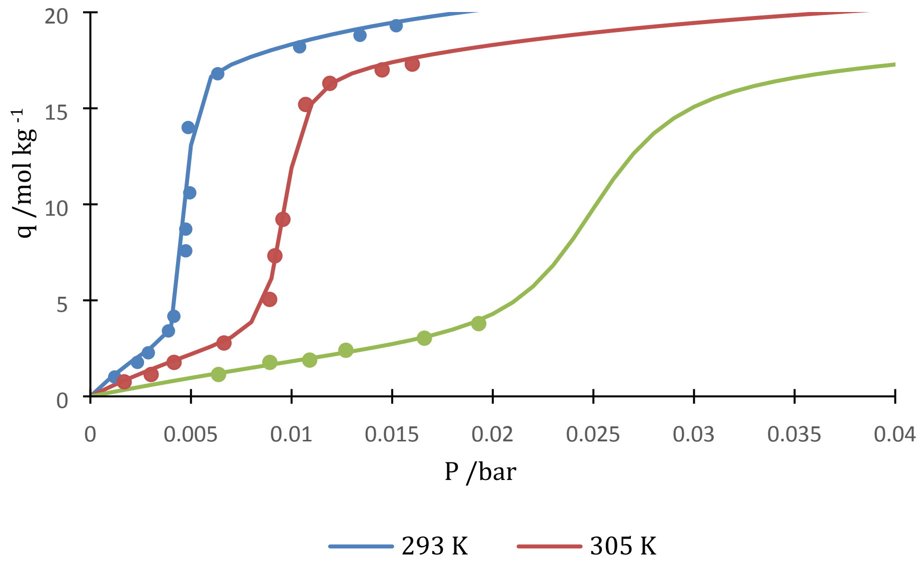

Figure SI. 2. MIL-125_NH$_2$ (Ti) dataset fitting to the Ising-Langmuir isotherm.[12,14]

Table SI. 2. Ising-Langmuir isotherm parameters for water adsorption in MIL-125_NH$_2$ (Ti). [12,14]

Parameter	Value
$q_{sat\ I}$ /mol kg^{-1}	13.0
$q_{sat\ L}$ /mol kg^{-1}	11.0
$K_{\infty,\ I}$ /bar^{-1}	3.81×10^{-6}
$K_{\infty,0}$ /bar^{-1}	0.299
$K_{\infty,L}$ /bar^{-1}	2.14×10^{-6}
$-\Delta H_I$ /kJ mol^{-1}	43.4
$-\Delta H_0$ /kJ mol^{-1}	0.0
$-\Delta H_L$ /kJ mol^{-1}	42.9

MIL-160 (Al)

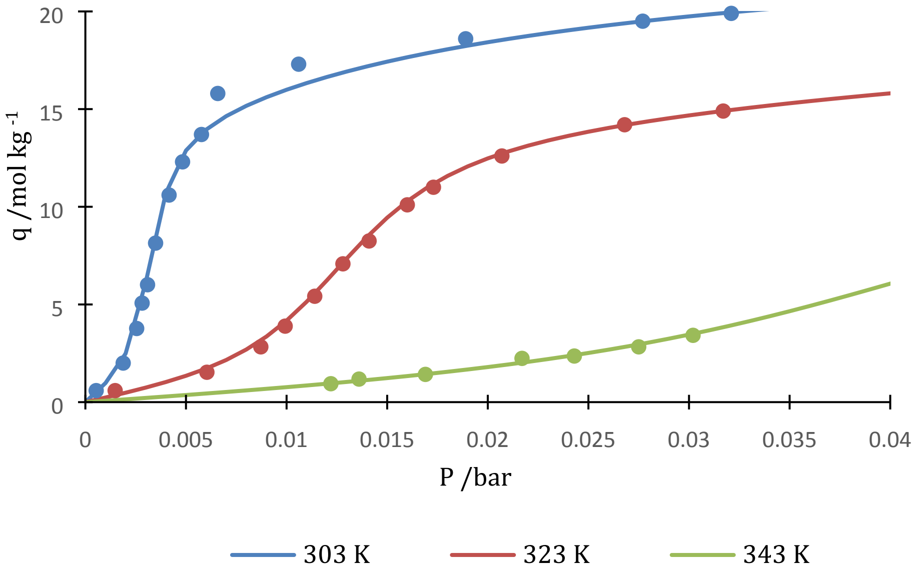

Figure SI. 3. MIL-160 (Al) dataset fitting to the Ising-Langmuir isotherm. [13,14]

Table SI. 3. Ising-Langmuir isotherm parameters for water adsorption in MIL-160 (Al) [13,14]

Parameter	Value

$q_{sat\ I}$ /mol kg^{-1}	11.3
$q_{sat\ L}$ /mol kg^{-1}	13.2
$K_{\infty,\ I}$ /bar^{-1}	5.24×10^{-8}
$K_{\infty,0}$ /bar^{-1}	2.81×10^{-7}
$K_{\infty,L}$ /bar^{-1}	7.08×10^{-9}
$-\Delta H_I$ /kJ mol^{-1}	56.6
$-\Delta H_0$ /kJ mol^{-1}	43.9
$-\Delta H_L$ /kJ mol^{-1}	57.6

CAU-10

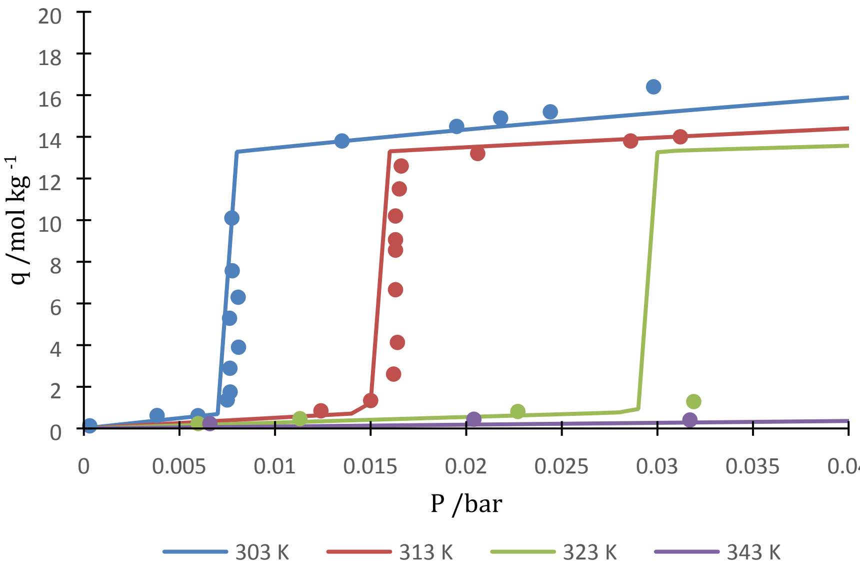

Figure SI. 4. CAU-10 dataset fitting to the Ising-Langmuir isotherm.[14]

Table SI. 4. Ising-Langmuir isotherm parameters for water adsorption in CAU-10.[14]

Parameter	Value
$q_{sat\ I}$ /mol kg^{-1}	12.5
$q_{sat\ L}$ /mol kg^{-1}	20.2
$K_{\infty,\ I}$ /bar^{-1}	2.99×10^{-8}

$K_{\infty,o}$ /bar^{-1}	8.50×10^{-9}
$K_{\infty,L}$ /bar^{-1}	5.25×10^{-9}
$-\Delta H_I$ /kJ mol^{-1}	56.0
$-\Delta H_0$ /kJ mol^{-1}	24.0
$-\Delta H_L$ /kJ mol^{-1}	52.1

Al-FUM

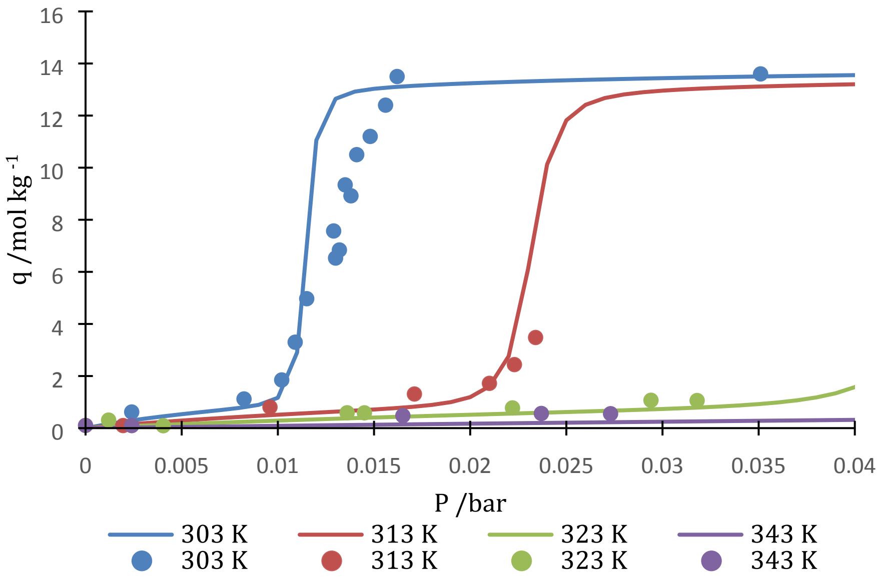

Figure SI. 5. Al-FUM dataset fitting to the Ising-Langmuir isotherm.[14]

Table SI. 5. Ising-Langmuir isotherm parameters for water adsorption in Al-FUM.[14]

Parameter	Value
$q_{sat\ I}$ /mol kg^{-1}	1.95
$q_{sat\ L}$ /mol kg^{-1}	12.1
$K_{\infty,\ I}$ /bar^{-1}	2.45×10^{-8}
$K_{\infty,o}$ /bar^{-1}	1.54×10^{-7}
$K_{\infty,L}$ /bar^{-1}	3.92×10^{-9}
$-\Delta H_I$ /kJ mol^{-1}	55.4

$-\Delta H_0$ /kJ mol^{-1}	32.0
$-\Delta H_L$ /kJ mol^{-1}	59.1

Zeolite 3A

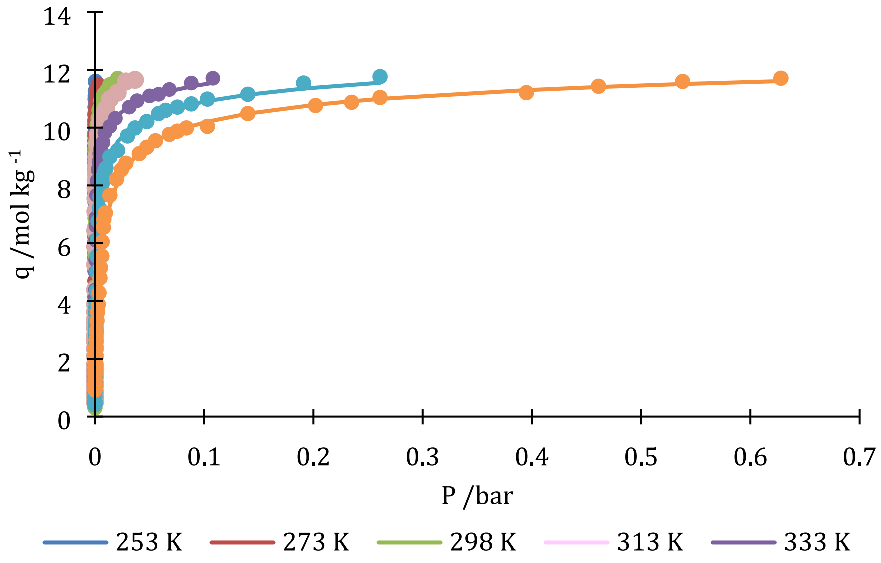

Figure SI. 6. Dataset fitting to the Ising-Langmuir isotherm for Zeolite 3A (data collected from Llano-Restrepo and Mosquera (2009) [15]*).*

Table SI. 6. Ising-Langmuir parameters for water adsorption in Zeolite 3A.

Parameter	Value
$q_{sat\ I}$ /mol kg^{-1}	5.60
$q_{sat\ L}$ /mol kg^{-1}	7.08
$K_{\infty,\ I}$ /bar^{-1}	4.48 x 10^{-6}
$K_{\infty,0}$ /bar^{-1}	2.21 x 10^{-6}
$K_{\infty,L}$ /bar^{-1}	1.79 x 10^{-6}
$-\Delta H_I$ /kJ mol^{-1}	52.6
$-\Delta H_0$ /kJ mol^{-1}	64.3
$-\Delta H_L$ /kJ mol^{-1}	55.9

Zeolite 4A

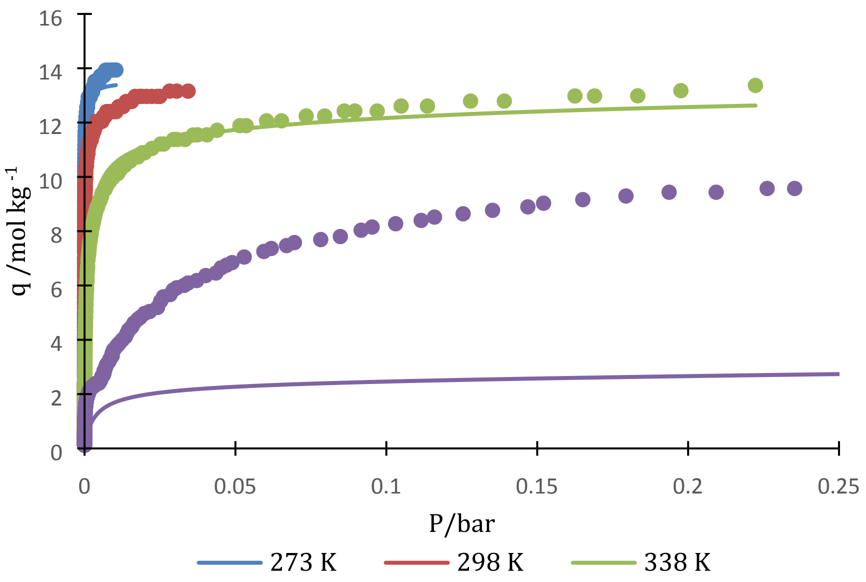

Figure SI. 7. Dataset fitting to the Ising-Langmuir isotherm for Zeolite 4A (data collected from Morris (1968) [16]*).*

Table SI. 7. Ising-Langmuir parameters for water adsorption in Zeolite 4A.

Parameter	Value
$q_{sat\ I}$ /mol kg^{-1}	5.38
$q_{sat\ L}$ /mol kg^{-1}	8.15
$K_{\infty,\ I}$ /bar^{-1}	5.64 x 10^{-5}
$K_{\infty,o}$ /bar^{-1}	1.14
$K_{\infty,L}$ /bar^{-1}	3.55 x 10^{-7}
$-\Delta H_I$ /kJ mol^{-1}	48.2
$-\Delta H_0$ /kJ mol^{-1}	33.3
$-\Delta H_L$ /kJ mol^{-1}	60.8

In this case, the fitting failed to predict the data from the isotherm at 422 K. To ensure the fitting could fit higher temperatures, data from another dataset [17] (which as shown a good concordance with the data used for the fitting) was compared with the result of the fitted isotherm equation.

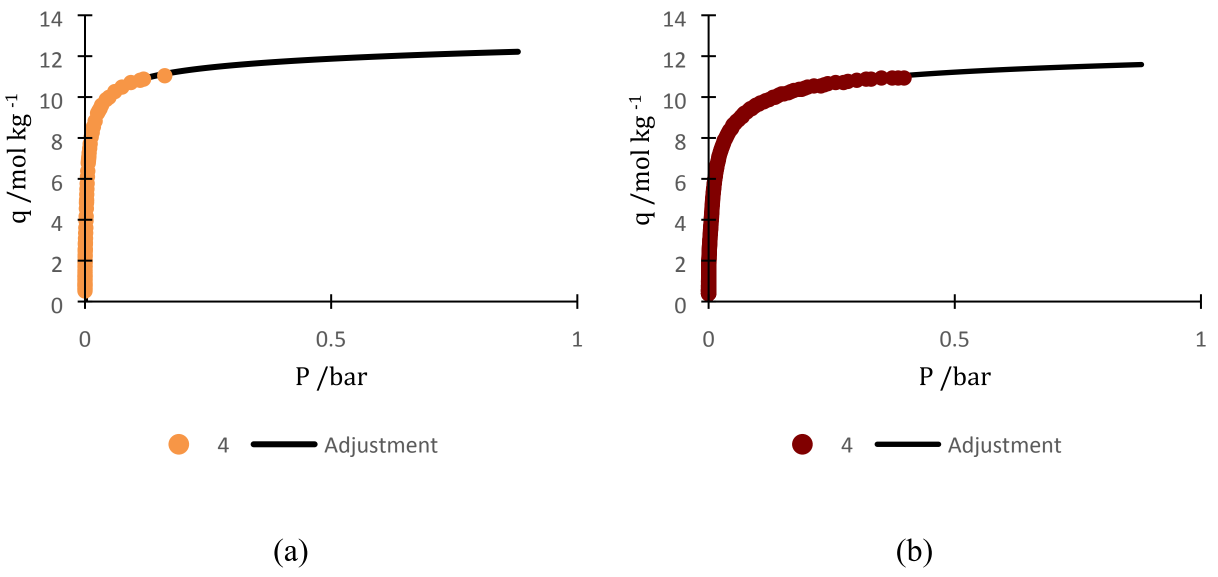

Figure SI. 8. Comparison of the alternative dataset with the curve obtained for 373 K (a) and 393 K (b).

Zeolite 5A

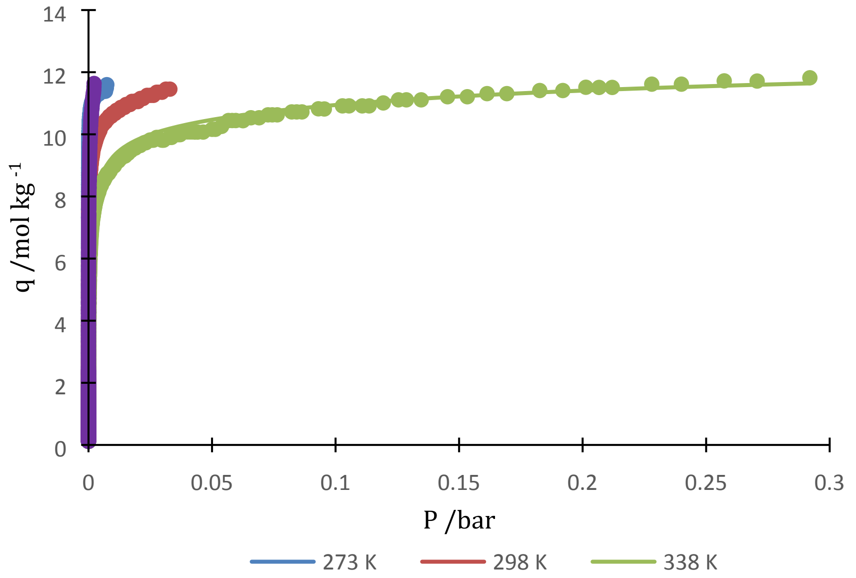

Figure SI. 9. Dataset 3 fitting to the Ising-Langmuir isotherm for Zeolite 5A (data collected from Morris (1968) [16]).

Table SI. 8. Ising-Langmuir parameters for water adsorption in Zeolite 5A.

Parameter	Value
$q_{sat\ I}$ /mol kg^{-1}	5.19
$q_{sat\ L}$ /mol kg^{-1}	6.22
$K_{\infty,\ I}$ /bar^{-1}	1.47 x 10^{-5}
$K_{\infty,0}$ /bar^{-1}	1.39 x 10^{-2}
$K_{\infty,L}$ /bar^{-1}	1.01 x 10^{-6}
$-\Delta H_I$ /kJ mol^{-1}	53.6
$-\Delta H_0$ /kJ mol^{-1}	47.0
$-\Delta H_L$ /kJ mol^{-1}	59.4

Zeolite 13 X

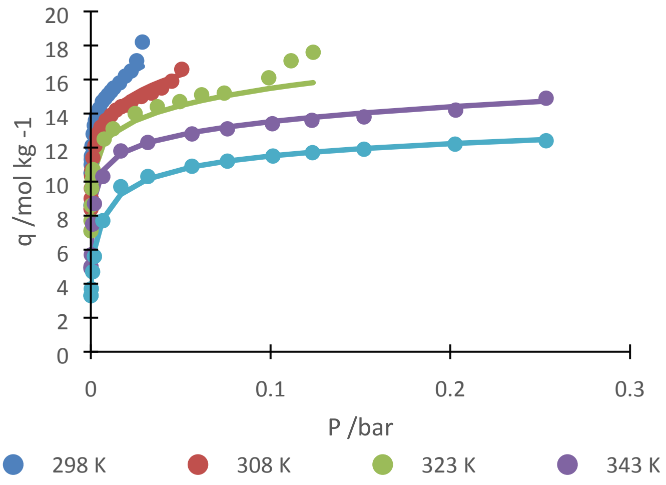

Figure SI. 10. Dataset fitting to the Ising-Langmuir isotherm for Zeolite 13X (data collected from Son et al. (2019) [18]).

Table SI. 9. Ising-Langmuir parameters for water adsorption in Zeolite 13 X.

Parameter	Value
$q_{sat\ I}$ /mol kg^{-1}	15.65
$q_{sat\ L}$ /mol kg^{-1}	4.30
$K_{\infty,\ I}$ /bar^{-1}	1.81 x 10^{-9}
$K_{\infty,0}$ /bar^{-1}	1.38 x 10^{-8}
$K_{\infty,L}$ /bar^{-1}	8.14 x 10^{-16}
$-\Delta H_I$ /kJ mol^{-1}	67.7
$-\ \Delta H_0$ /kJ mol^{-1}	69.7
$-\ \Delta H_L$ /kJ mol^{-1}	140.0

AQSOA FAM-Z02

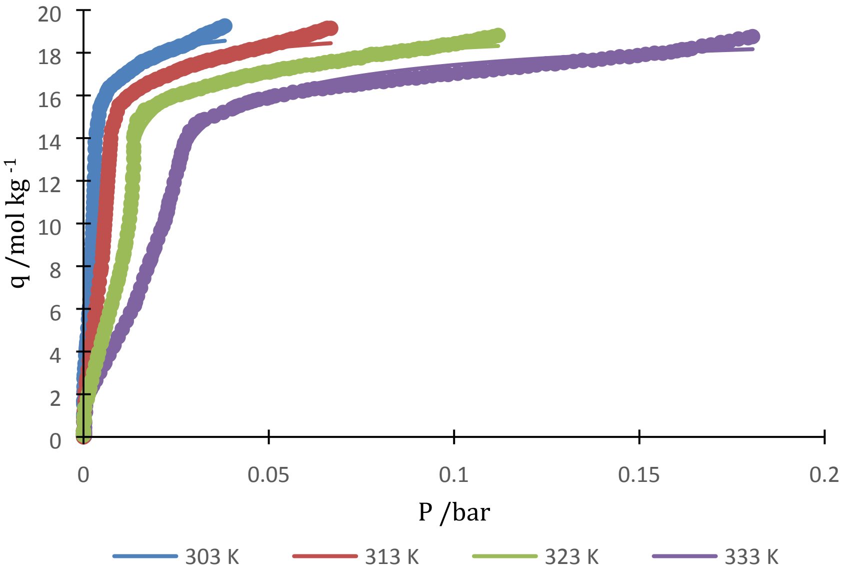

Figure SI. 11. Dataset 4 fitting to the Ising-Langmuir isotherm for AQSOA FAM-Z02 (data collected from Fasano et al. (2019) [19]*).*

Table SI. 10. Ising-Langmuir parameters for water adsorption in AQSOA FAM-Z02.

Parameter	Value
$q_{sat\ I}$ /mol kg^{-1}	6.11
$q_{sat\ L}$ /mol kg^{-1}	13.11
$K_{\infty,\ I}$ /bar^{-1}	3.05 x 10^{-8}
$K_{\infty,0}$ /bar^{-1}	0.41
$K_{\infty,L}$ /bar^{-1}	6.92 x 10^{-8}
$-\Delta H_I$ /kJ mol^{-1}	58.3
$-\ \Delta H_0$ /kJ mol^{-1}	0
$-\ \Delta H_L$ /kJ mol^{-1}	57.2

Isosteric heat of adsorption

The isosteric heat of adsorption was calculated for certain values of q according to the Clausius-Clapeyron equation (Equation SI.18).

$$(-\Delta H_{ads}) = RT^2\left(\frac{\delta \ln P}{\delta T}\right)\Bigg|_{q\,=\,const} \tag{SI.18}$$

Considering that $(-\Delta H_{ads})$ is independent of the temperature, then its value can be obtained through a linear fitting for each q, according to Equation SI.19.

$$\ln P = -\frac{(-\Delta H_{ads})}{R}\frac{1}{T} + G \tag{SI.19}$$

Where G is the integration constant.

After obtaining those points, a polynomial fitting was made for one or more branches of the function that would describe the obtained values. Equation SI.20 describes the polynomial fitting made.

$$(-\Delta H_{ads}) = a_6\ q^6 + a_5\ q^5 + a_4\, q^4 + a_3\, q^3 + a_2\, q^2 + a_1\, q + a_0 \tag{SI.20}$$

With $(-\Delta H_{ads})$ in J mol^{-1}, q in mol kg^{-1} and a_6, a_5,a_4, a_3, a_2, a_1 and a_0 being the polynomial coefficients in the corresponding units.

In the following subsections, the plots of the isosteric heat of adsorption vs. the amount of water adsorbed are presented, as well as the values of the coefficients for the polynomial fitting in each branch. In the points where no data was collected, the value was considered constant and equal to the isosteric heat of adsorption of the nearest point collected.

MIL-100 (Fe)

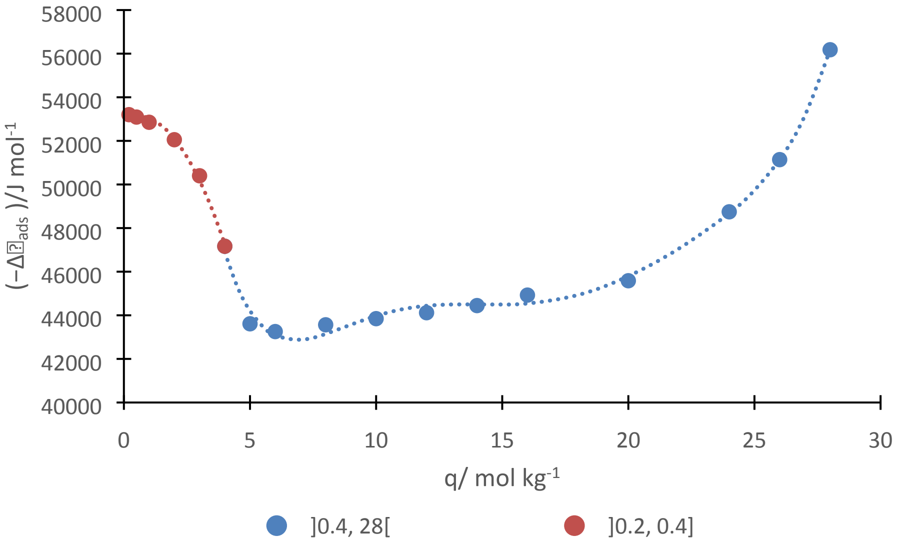

Figure SI. 12. Adjustment of isosteric heat of adsorption in function of the adsorbed amount for MIL-100 (Fe).

Table SI. 11. Parameters of the polynomial fitting of isosteric heat of adsorption for MIL-100 (Fe).

/mol kg^{-1}							
[0,0.2]							53 202
]0.2,0.4]					-493.90	549.00	52 975
]0.4, 28[	5.02 x 10^{-3}	- 0.51	20.53	-423.36	4 635	-25 162	95 723
[28, ∞[							56 179

MIL-125 _NH_2 (Ti)

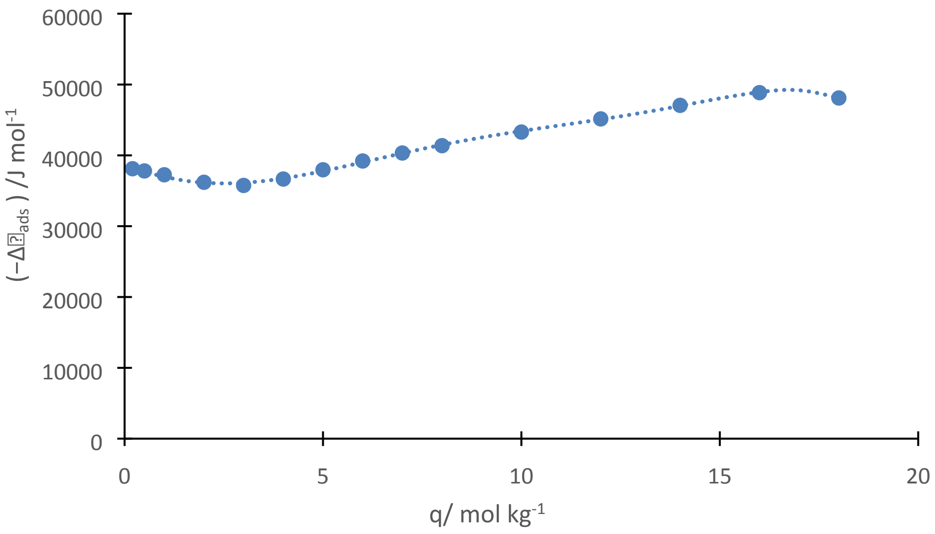

Figure SI. 13. Adjustment of isosteric heat of adsorption in function of the adsorbed amount for MIL-125_NH_2 (Ti).

Table SI. 12. Parameters of the polynomial fitting of isosteric heat of adsorption for MIL-125_NH_2 (Ti).

/mol kg^{-1}							
[0,0.2]							38 114
]0.2,18[	-1.37 x10^{-2}	0.61	-8.40	18.24	459.72	-2 231	38 736
[18, ∞[							48 100

MIL-160 (Al)

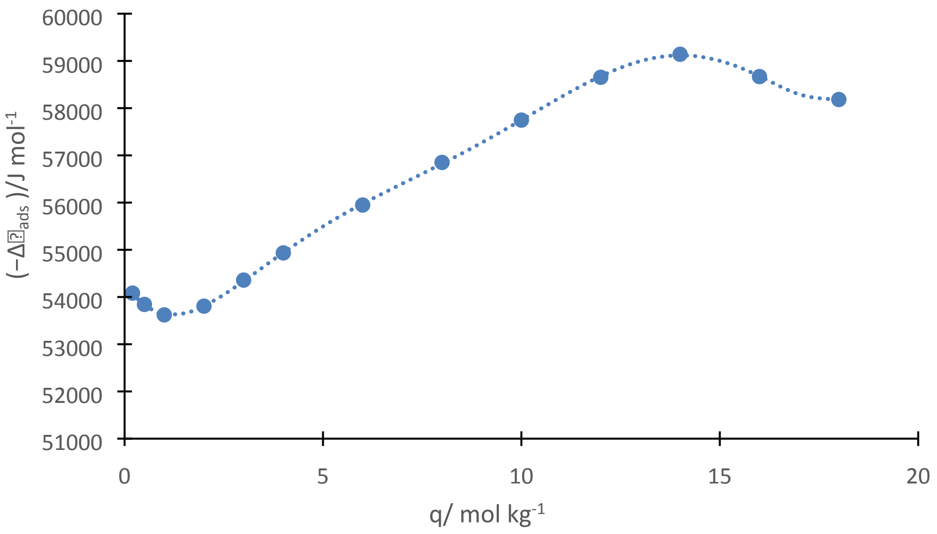

Figure SI. 14. Adjustment of isosteric heat of adsorption in function of the adsorbed amount for MIL-160 (Al).

Table SI. 13. Parameters of the polynomial fitting of isosteric heat of adsorption for MIL-160 (Al).

/mol kg^{-1}							
[0,0.2]							54 083
]0.2,18[	1.31 x10^{-2}	-0.73	15.49	-158.46	803.58	-1 342	54 321
[18, ∞ [							58 183

CAU-10

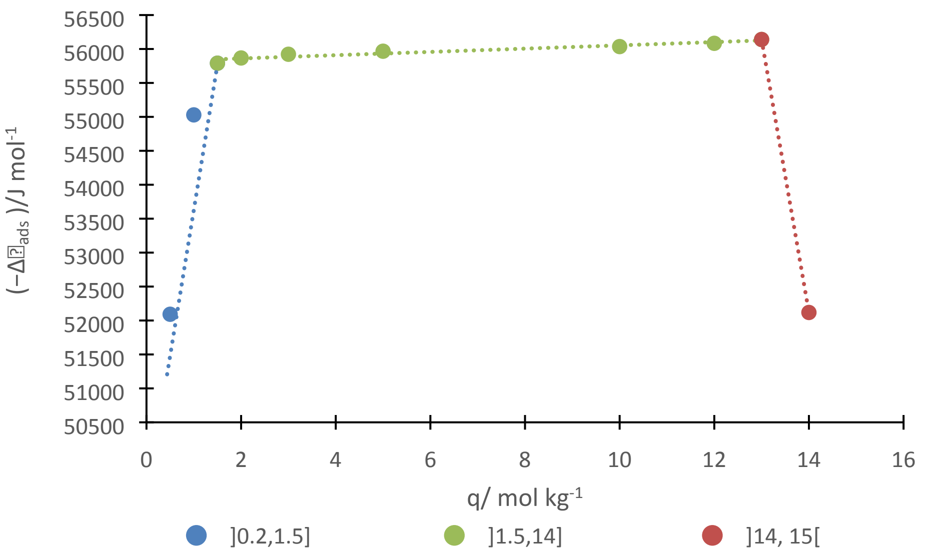

Figure SI. 15. Adjustment of isosteric heat of adsorption in function of the adsorbed amount for CAU-10.

Table SI. 14. Parameters of the polynomial fitting of isosteric heat of adsorption for CAU-10.

/mol kg^{-1}							
[0,0.2]							52 096
]0.2,1.5]						3 698	50 606
]1.5,14]							55 972
]14, 15[						-4 021	108 421
[15, ∞ [							52 101

Al-FUM

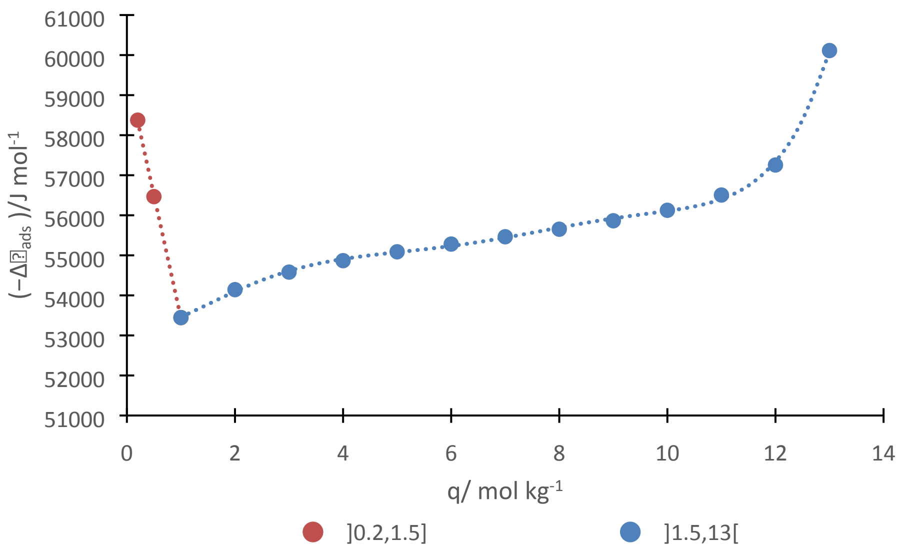

Figure SI. 16. Adjustment of isosteric heat of adsorption in function of the adsorbed amount for Al-FUM.

Table SI. 15. Parameters of the polynomial fitting of isosteric heat of adsorption for Al-FUM.

/mol kg^{-1}							
[0,0.2]							58 375
]0.2,1.5]						-6 151	59 581
]1.5,13[	7.82 x 10^{-2}	-2.81	38.32	-242.85	665.21	-150.4	53 151
[13, ∞ [							60 114

Zeolite 3A

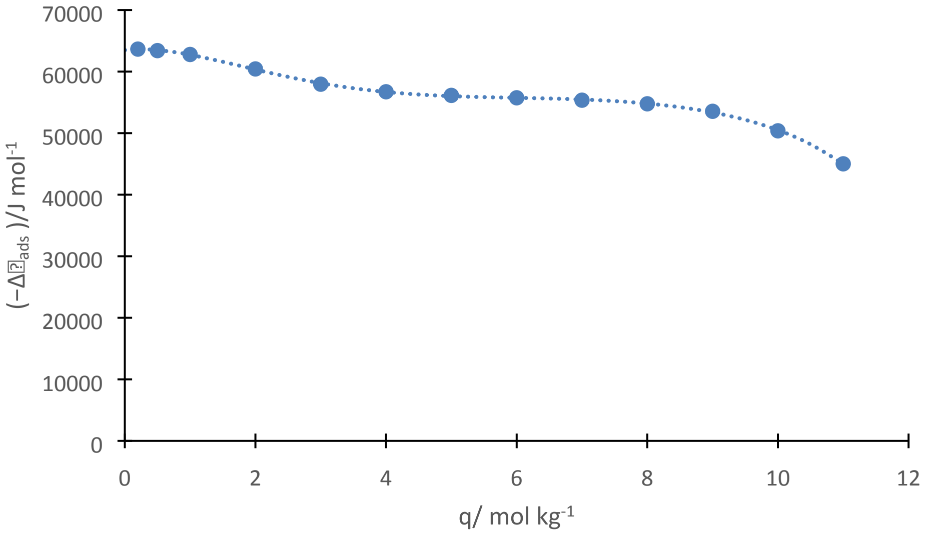

Figure SI. 17. Adjustment of isosteric heat of adsorption in function of the adsorbed amount for Zeolite 3A.

Table SI. 16. Parameters of the polynomial fitting of isosteric heat of adsorption for Zeolite 3A.

/mol kg^{-1}							
[0,0.2]							63 654
]0.2,11]	-0.20	7.33	-109.91	796.34	-2 543	1 093	63 493
[11, ∞ [							45 016

Zeolite 4A

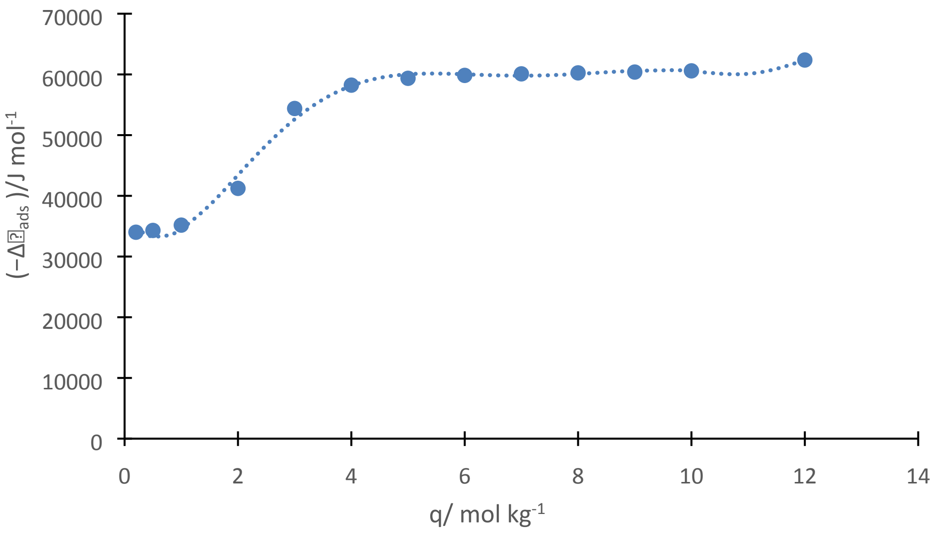

Figure SI. 18. Adjustment of isosteric heat of adsorption in function of the adsorbed amount for Zeolite 4A.

Table SI. 17. Parameters of the polynomial fitting of isosteric heat of adsorption for Zeolite 4A.

/mol kg^{-1}							
[0,0.2]							34 006
]0.2,12]	1.04	-41.9	650.5	-4 806	16 178	-14 528	37 087
[12, ∞ [							62 372

Zeolite 5A

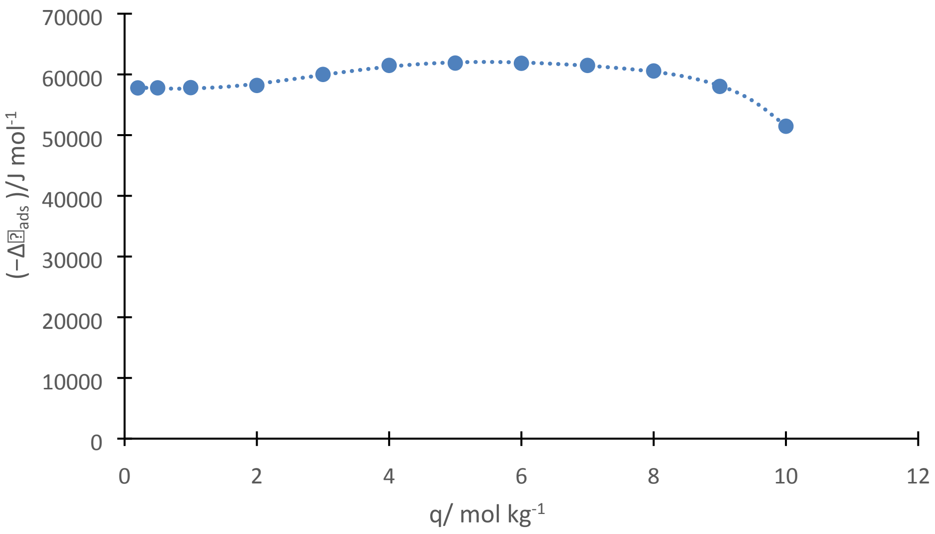

Figure SI. 19. Adjustment of isosteric heat of adsorption in function of the adsorbed amount for Zeolite 5A.

Table SI. 18. Parameters of the polynomial fitting of isosteric heat of adsorption for Zeolite 5A.

/mol kg^{-1}							
[0,0.2]							57 779
]0.2,10]	-0.47	11.69	-99.27	269.51	289.87	-812.36	58 018
[10, ∞ [							51 465

Zeolite 13 X

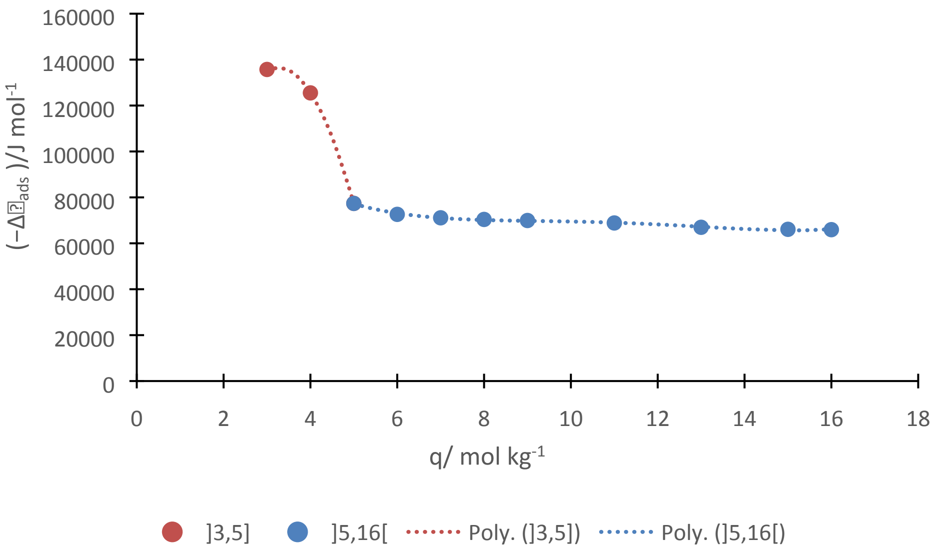

Figure SI. 20.Adjustment of isosteric heat of adsorption in function of the adsorbed amount for Zeolite 13X.

Table SI. 19. Parameters of the polynomial fitting of isosteric heat of adsorption for Zeolite 13X.

/mol kg^{-1}							
[0,3]							135 715
]3,5]					-18 965	122 552	-61 251
]5,16[			7.21	-320.07	5 172	-36 622	166 438
[16, ∞ [							65 941

AQSOA FAM-Z02

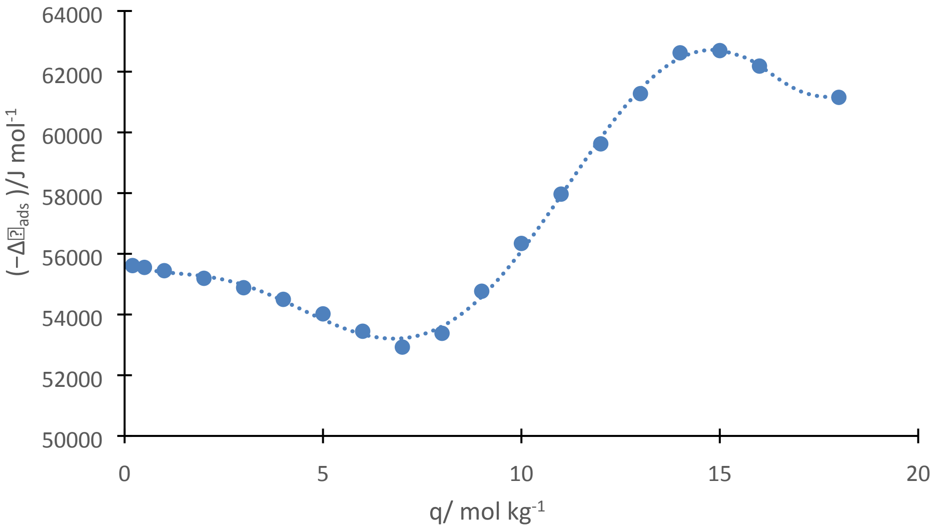

Figure SI. 21. Adjustment of isosteric heat of adsorption in function of the adsorbed amount for AQSOA FAM-Z02.

Table SI. 20. Parameters of the polynomial fitting of isosteric heat of adsorption for AQSOA FAM-Z02.

/mol kg^{-1}							
[0,0.2]							55 612
]0.2,18]	3.2 x 10^{-2}	-1.59	27.88	-198.29	547.15	-757.52	55 778
[18, ∞ [							61 155

Specific heat capacity of adsorbents

Table SI. 21. Specific heat capacity of adsorbents.

Material	Value or function /J kg^{-1} K^{-1}	Temperature range /K
MIL-100(Fe) [20]	990	-
MIL-125_NH_2 (Ti) [21]	890	]0, 308]
	$4.46\,T - 484.15$	]308, 373[
	1 180	[373, ∞[
MIL-160(Al) [22]	1 117	]0, 297.8]
	$0.97\,T + 824.36$	]297.8, 343[
	1 160	[343, ∞[
CAU-10 [23]	1 300	]0, 298]
	$6.87\,T - 780.58$	]298, 352.9]
	$-10\,T + 5179$	]352.9, 362.9]
	$2.42\,T + 656.27$	]362.9, 423[
	1 700	[423, ∞[
Al-FUM [24]	1 050	]0, 323]
	$1\,262 + 3\,(T - 273) + \frac{17\,379}{(T - 273)^2}$	]323, 418[
	1 350	[418, ∞[
Zeolite 3A [25]	1045	-
Zeolite 4A [26]	920	-

Zeolite 5A [27]	921.1	-
Zeolite 13X [28]	1004.46	]0, 313.61]
	$1097.44 + 3.44\ (T - 373) - 0.0029\ (T^2 - 373^2)$	]313.61, 452.14[
	1185.55	[452.14, ∞]
AQSOA Z02-FAM[29]	822	]0, 303]
	$2\ T + 216$	]303, 363[
	942	[363, ∞[

Particle distribution evaluation

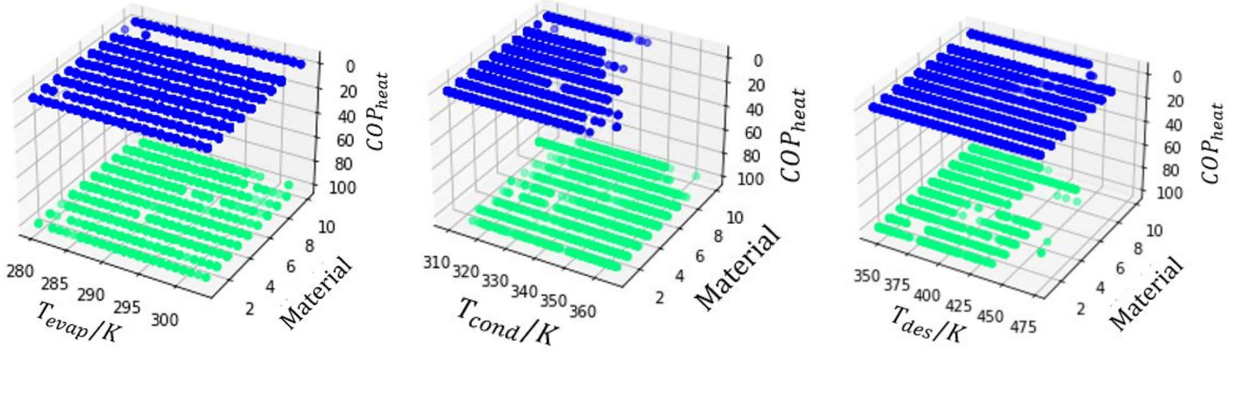

(a)

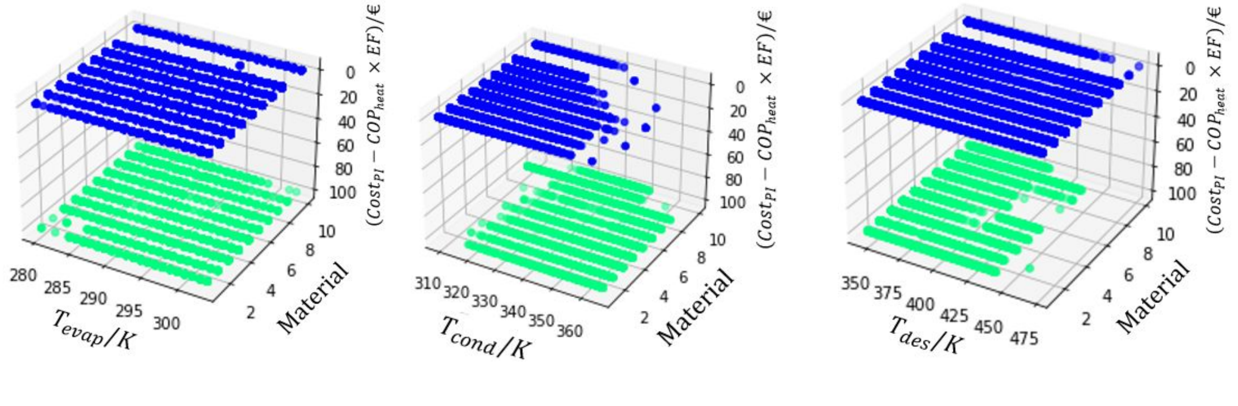

(b)

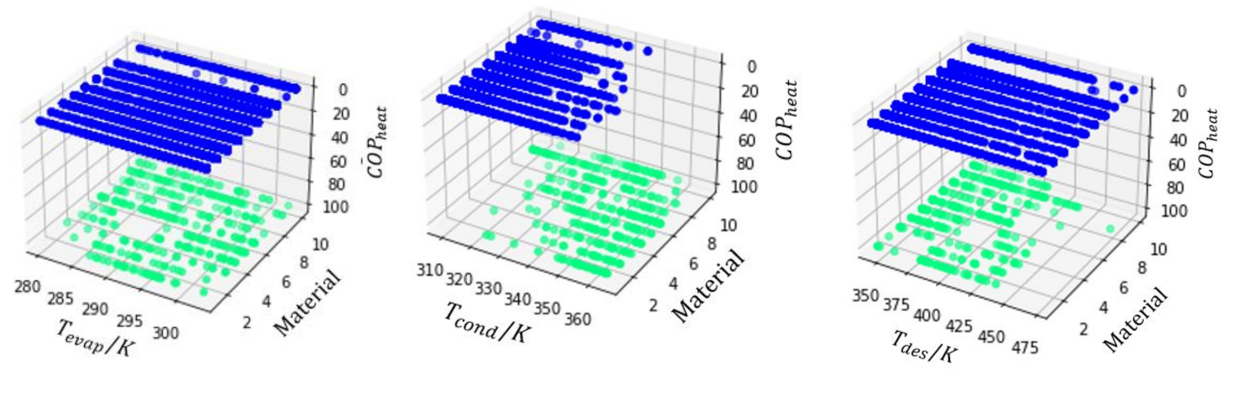

(c)

Figure SI. 22. Particle distribution during the PSO implementation in maximization of COP_{heat} (a), minimization of $(Cost_{PI} - COP_{heat} \times EF)$ (b), and multi-objective problem (c).

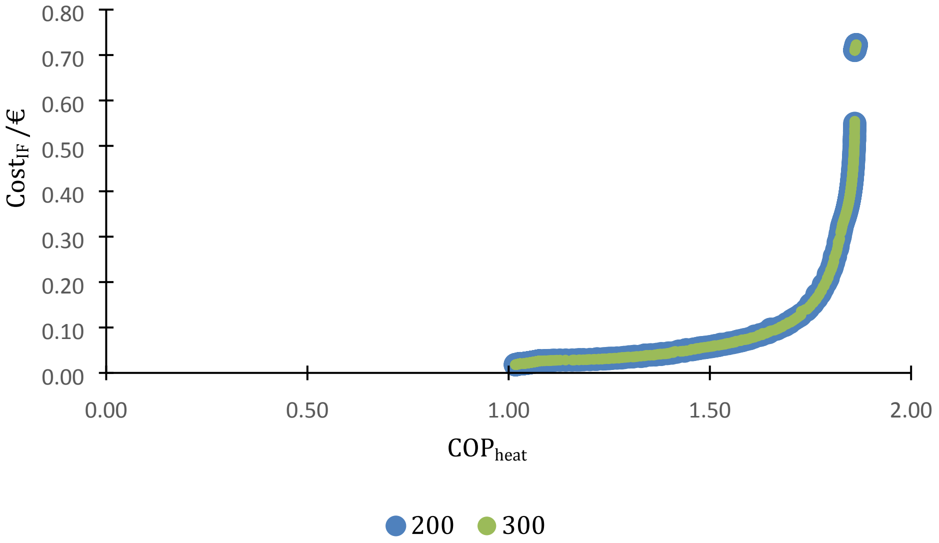

Figure SI. 23. Pareto Front for different N_{it_max}.

Mapping the temperatures

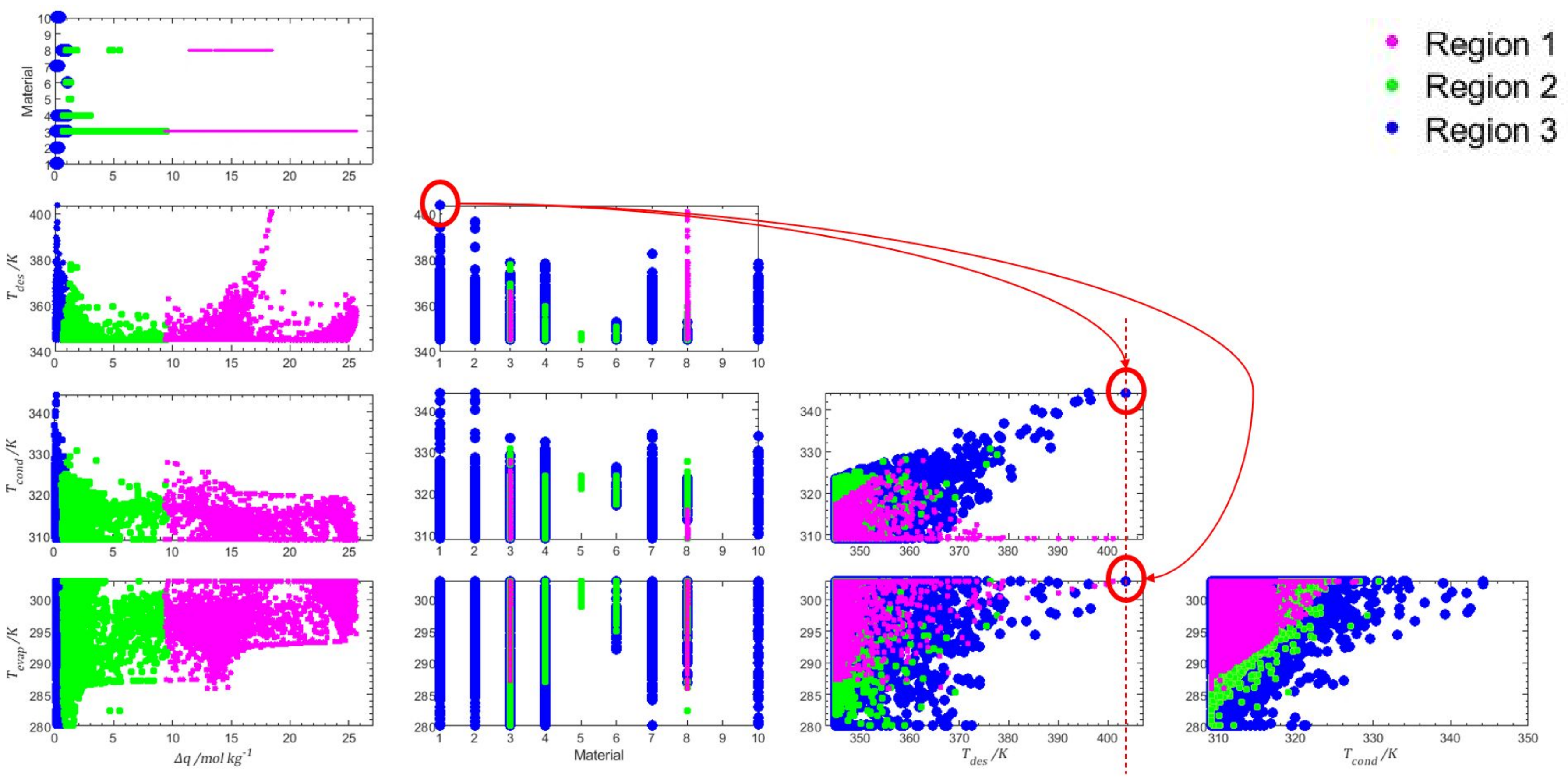

Figure SI. 24. Mapping the temperature set and the material corresponding to T_{des} of ≈ 400 K for Region 3.

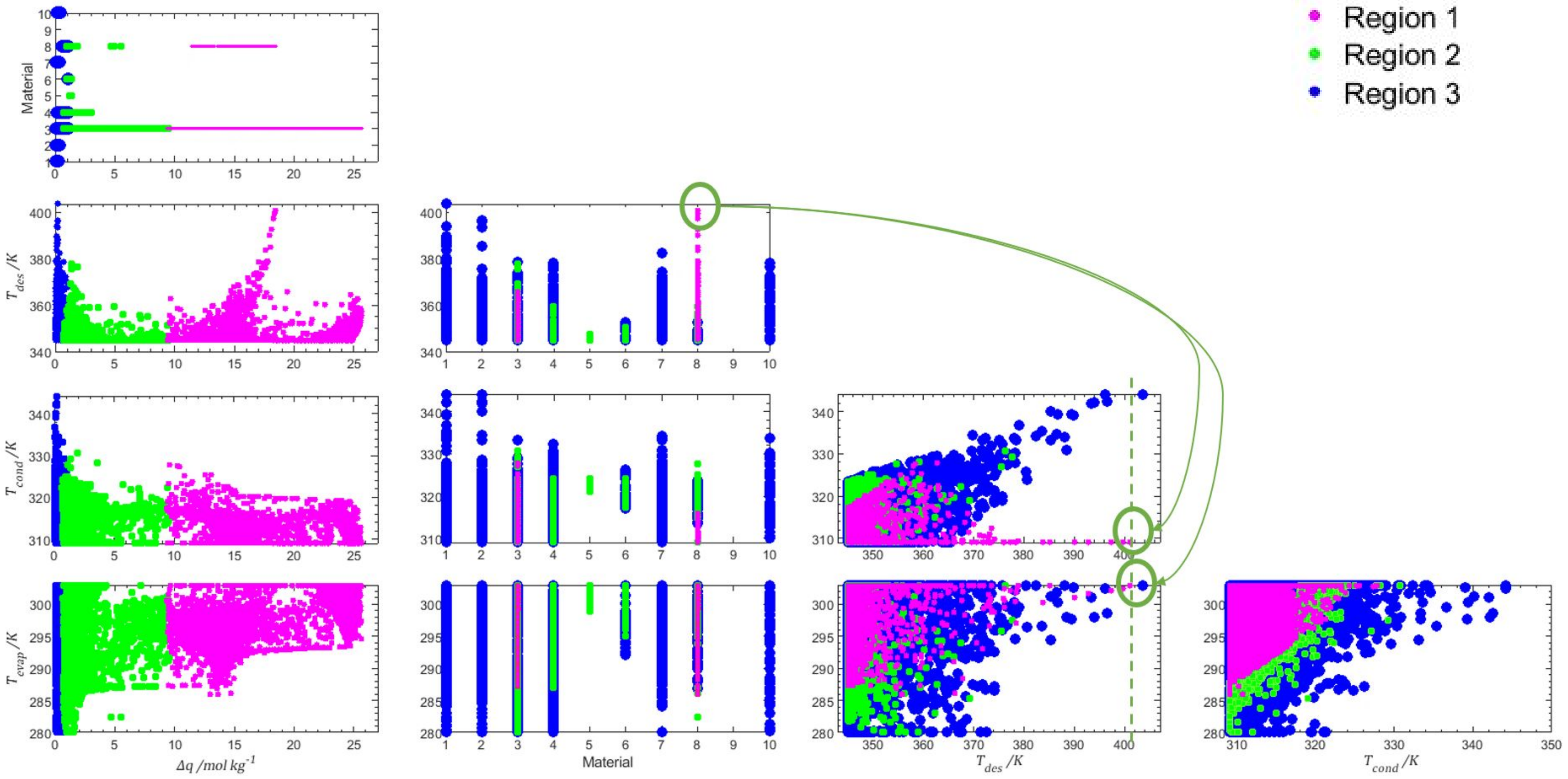

Figure SI. 25. Mapping the temperature set and the material corresponding to T_{des} of ≈ 400 K for Region 1.

References

(1) Demir, H.; Mobedi, M.; Ülkü, S. A Review on Adsorption Heat Pump: Problems and Solutions. *Renewable and Sustainable Energy Reviews* **2008**, *12* (9), 2381–2403. https://doi.org/10.1016/j.rser.2007.06.005.

(2) Dias, J. M. S.; Costa, V. A. F. Adsorption Heat Pumps for Heating Applications: A Review of Current State, Literature Gaps and Development Challenges. *Renewable and Sustainable Energy Reviews* **2018**, *98*, 317–327. https://doi.org/10.1016/j.rser.2018.09.026.

(3) Boman, D.; Raymond, A.; Garimella, S. *Adsorption Heat Pumps: Fundamentals and Applications*; 2021. https://doi.org/10.1007/978-3-030-72180-0.

(4) Ülkü, S. Adsorption Heat Pumps. *Journal of Heat Recovery Systems* **1986**, *6* (4), 277–284. https://doi.org/10.1016/0198-7593(86)90113-X.

(5) Pinheiro, J. M.; Salústio, S.; Rocha, J.; Valente, A. A.; Silva, C. M. Adsorption Heat Pumps for Heating Applications. *Renewable and Sustainable Energy Reviews* **2020**, *119*, 109528. https://doi.org/10.1016/j.rser.2019.109528.

(6) Critoph, R. E. Performance Limitations of Adsorption Cycles for Solar Cooling. *Solar Energy* **1988**, *41* (1), 21–31. https://doi.org/10.1016/0038-092X(88)90111-9.

(7) Aristov, Yu. I.; Tokarev, M. M.; Sharonov, V. E. Universal Relation between the Boundary Temperatures of a Basic Cycle of Sorption Heat Machines. *Chem Eng Sci* **2008**, *63* (11), 2907–2912. https://doi.org/10.1016/j.ces.2008.03.011.

(8) Liu, Z.; Li, W.; Cai, S.; Tu, Z.; Luo, X.; Li, S. Screening Versatile Water/Adsorbent Working Pairs for Wide Operating Conditions of Adsorption Heat Pumps. *Sustainable Energy Fuels* **2022**, *6* (2), 309–319. https://doi.org/10.1039/D1SE01819A.

(9) NIST. *Liquid Phase Heat Capacity (Shomate Equation) for water*. https://webbook.nist.gov/cgi/cbook.cgi?ID=C7732185&Mask=2#Thermo-Condensed (accessed 2022-04-15).

(10) NIST. *Antoine Equation Parameters for water*. https://webbook.nist.gov/cgi/cbook.cgi?ID=C7732185&Mask=4#Thermo-Phase (accessed 2022-04-15).

(11) Silva, M. P.; Ribeiro, A. M.; Silva, C. G.; Ho Cho, K.; Lee, U.-H.; Faria, J. L.; Loureiro, J. M.; Chang, J.-S.; Rodrigues, A. E.; Ferreira, A. Atmospheric Water Harvesting on MIL-100(Fe) upon a Cyclic Adsorption Process. *Sep Purif Technol* **2022**, *290*, 120803. https://doi.org/10.1016/j.seppur.2022.120803.

(12) Silva, M. P.; Ribeiro, A. M.; Silva, C. G.; Narin, G.; Nogueira, I. B. R.; Lee, U.-H.; Faria, J. L.; Loureiro, J. M.; Chang, J.-S.; Rodrigues, A. E.; Ferreira, A. Water

Vapor Harvesting by a (P)TSA Process with MIL-125(Ti)_NH2 as Adsorbent. *Sep Purif Technol* **2020**, *237*, 116336. https://doi.org/10.1016/j.seppur.2019.116336.

(13) Silva, M. P.; Ribeiro, A. M.; Silva, C. G.; Nogueira, I. B. R.; Cho, K.-H.; Lee, U.-H.; Faria, J. L.; Loureiro, J. L.; Chang, J.-S.; Rodrigues, A. E.; Ferreira, A. MIL-160(Al) MOF's Potential in Adsorptive Water Harvesting. *Adsorption* **2021**, *27* (2), 213–226. https://doi.org/10.1007/s10450-020-00286-5.

(14) Silva, M. Water Harvesting by Adsorption Based Processes on MOFs. Ph.D. Thesis, Universidade do Porto, 2021. https://hdl.handle.net/10216/133872.

(15) Llano-Restrepo, M.; Mosquera, M. A. Accurate Correlation, Thermochemistry, and Structural Interpretation of Equilibrium Adsorption Isotherms of Water Vapor in Zeolite 3A by Means of a Generalized Statistical Thermodynamic Adsorption Model. *Fluid Phase Equilib* **2009**, *283* (1), 73–88. https://doi.org/10.1016/j.fluid.2009.06.003.

(16) Morris, B. Heats of Sorption in the Crystalline Linde-A Zeolite-Water Vapor System. *J Colloid Interface Sci* **1968**, *28* (1), 149–155.

(17) Grace. *Process Adsorbents*. https://grace.com/industries/general-industrial/process-adsorbents/ (accessed 2022-03-22).

(18) Son, K. N.; Richardson, T.-M. J.; Cmarik, G. E. Equilibrium Adsorption Isotherms for H2O on Zeolite 13X. *J Chem Eng Data* **2019**, *64* (3), 1063–1071. https://doi.org/10.1021/acs.jced.8b00961.

(19) Fasano, M.; Falciani, G.; Brancato, V.; Palomba, V.; Asinari, P.; Chiavazzo, E.; Frazzica, A. Atomistic Modelling of Water Transport and Adsorption Mechanisms in Silicoaluminophosphate for Thermal Energy Storage. *Appl Therm Eng* **2019**, *160*, 114075. https://doi.org/10.1016/j.applthermaleng.2019.114075.

(20) Hussein, E. Numerical and Experimental Evaluation of Advanced Metal-Organic Framework Materials for Adsorption Heat Pumps. Ph.D. thesis, University of Birmingham, 2018.

(21) Gordeeva, L. G.; Solovyeva, M. v; Aristov, Y. I. NH2-MIL-125 as a Promising Material for Adsorptive Heat Transformation and Storage. *Energy* **2016**, *100*, 18–24. https://doi.org/https://doi.org/10.1016/j.energy.2016.01.034.

(22) Cui, S.; Marandi, A.; Lebourleux, G.; Thimon, M.; Bourdon, M.; Chen, C.; Severino, M. I.; Steggles, V.; Nouar, F.; Serre, C. Heat Properties of a Hydrophilic Carboxylate-Based MOF for Water Adsorption Applications. *Appl Therm Eng* **2019**, *161*, 114135. https://doi.org/10.1016/j.applthermaleng.2019.114135.

(23) Fröhlich, D.; Pantatosaki, E.; Kolokathis, P. D.; Markey, K.; Reinsch, H.; Baumgartner, M.; van der Veen, M. A.; de Vos, D. E.; Stock, N.; Papadopoulos, G. K.; Henninger, S. K.; Janiak, C. Water Adsorption Behaviour of CAU-10-H: A Thorough Investigation of Its Structure–Property Relationships. *J Mater Chem A Mater* **2016**, *4* (30), 11859–11869. https://doi.org/10.1039/C6TA01757F.

(24) Jahan, I.; Islam, Md. A.; Palash, M. L.; Rocky, K. A.; Rupam, T. H.; Saha, B. B. Experimental Study on the Influence of Metal Doping on Thermophysical Properties of Porous Aluminum Fumarate. *Heat Transfer Engineering* **2021**, *42* (13–14), 1132–1141. https://doi.org/10.1080/01457632.2020.1777005.

(25) Simo, M.; Sivashanmugam, S.; Brown, C. J.; Hlavacek, V. Adsorption/Desorption of Water and Ethanol on 3A Zeolite in Near-Adiabatic Fixed Bed. *Ind Eng Chem Res* **2009**, *48* (20), 9247–9260. https://doi.org/10.1021/ie900446v.

(26) Gorbach, A.; Stegmaier, M.; Eigenberger, G. Measurement and Modeling of Water Vapor Adsorption on Zeolite 4A—Equilibria and Kinetics. *Adsorption* **2004**, *10* (1), 29–46. https://doi.org/10.1023/B:ADSO.0000024033.60103.ff.

(27) Bakhtyari, A.; Mofarahi, M. Pure and Binary Adsorption Equilibria of Methane and Nitrogen on Zeolite 5A. *J Chem Eng Data* **2014**, *59* (3), 626–639. https://doi.org/10.1021/je4005036.

(28) Lu, X.; Wang, Y.; Estel, L.; Kumar, N.; Grénman, H.; Leveneur, S. Evolution of Specific Heat Capacity with Temperature for Typical Supports Used for Heterogeneous Catalysts. *Processes* **2020**, *8* (8). https://doi.org/10.3390/pr8080911.

(29) Kakiuchi, H.; Iwade, M.; Shimooka, S.; Ooshima, K.; Yamazaki, M.; Takewaki, T. Novel Zeolite Adsorbents and Their Application for AHP and Desiccant System. *IEA-Annex* **2005**, *17*.
